# Current Genetic Service Delivery Models for the Provision of Genetic Testing in Europe: A Systematic Review of the Literature

**DOI:** 10.3389/fgene.2019.00552

**Published:** 2019-06-19

**Authors:** Brigid Unim, Erica Pitini, Tyra Lagerberg, Giovanna Adamo, Corrado De Vito, Carolina Marzuillo, Paolo Villari

**Affiliations:** ^1^Department of Public Health and Infectious Diseases, Sapienza University of Rome, Rome, Italy; ^2^Better Value HealthCare, Ltd., Oxford, United Kingdom

**Keywords:** genetic services, delivery model, genetic test, predictive testing, genetic program, systematic review

## Abstract

**Background:** The provision of genetic services, along with research in the fields of genomics and genetics, has evolved in recent years to meet the increasing demand of consumers interested in prediction of genetic diseases and various inherited traits. The aim of this study is to evaluate genetic services in order to identify and classify delivery models for the provision of genetic testing in European and in extra-European countries.

**Methods:** A systematic review of the literature was conducted using five electronic resources. Inclusion criteria were that studies be published in English or Italian during the period 2000–2015 and carried out in European or extra-European countries (Canada, USA, Australia, or New Zealand).

**Results:** 148 genetic programs were identified in 117 articles and were delivered mostly in the UK (59, 40%), USA (35, 24%) or Australia (16, 11%). The programs were available nationally (66; 45%), regionally (49; 33%) or in urban areas (21, 14%). Ninety-six (64%) of the programs were integrated into healthcare systems, 48 (32.21%) were pilot programs and five (3%) were direct-to-consumer genetic services. The genetic tests offered were mainly for BRCA1/2 (59, 40%), Lynch syndrome (23, 16%), and newborn screening (18, 12%). Healthcare professionals with different backgrounds are increasingly engaged in the provision of genetic services. Based on which healthcare professionals have prominent roles in the respective patient care pathways, genetic programs were classified into five models: (i) the geneticists model; (ii) the primary care model; (iii) the medical specialist model; (iv) the population screening programs model; and (v) the direct-to-consumer model.

**Conclusions:** New models of genetic service delivery are currently under development worldwide to address the increasing demand for accessible and affordable services. These models require the integration of genetics into all medical specialties, collaboration among different healthcare professionals, and the redistribution of professional roles. An appropriate model for genetic service provision in a specific setting should ideally be defined according to the type of healthcare system, the genetic test provided within a genetic program, and the cost-effectiveness of the intervention. Only applications with proven efficacy and cost-effectiveness should be implemented in healthcare systems and made available to all citizens.

## Introduction

### Rationale

In genomic medicine, the transfer of genetic tests from research to clinical practice can be defined as stage three (T3) translational research, which “attempts to move evidence-based guidelines into health practice, through delivery, dissemination, and diffusion research” (Khoury et al., 2007). This process is influenced by factors inherent to research and delivery of healthcare, but also by external and commercial interests. One particular concern is the introduction, in both the public and private health sectors, of predisposition and predictive genetic tests for which sufficient evidence of analytical and clinical validity, clinical utility, and cost-effectiveness is lacking (Scheuner et al., 2008).

The scientific evidence on the effectiveness and cost-effectiveness of genetic tests is currently limited compared with the large number of tests available (National Academies of Sciences, Engineering, and Medicine, 2017). For instance, the National Institutes of Health Genetic Testing Registry reports more than 55,000 available genetic tests (National Library of Medicine, 2018) while the National Library of Medicine lists 457 professional practice guidelines, position statements, and recommendations on genetic tests that have been identified (MedGen, 2017). The difference between the number of genetic tests and the number of evidence-based records indicates that most tests have not been evaluated and highlights the need for evidence-based technology assessments and economic evaluations prior to introduction of genomics applications in clinical and public health practice.

### Objectives

The introduction of genomic tests in practice forms just one aspect of what constitutes an optimal genetic service delivery model, which is defined as the broad context within a public health genomics (PHG) framework in which genetic services are offered to individuals and families with or at risk of genetic disorders. In other words, a genetic service delivery model is a combination of personal healthcare services provided by healthcare professionals to individuals and families (i.e., diagnosis, treatment/management, and information), and public health services and functions (i.e., population screening, financing, policy development, workforce education, information/citizen empowerment, service evaluation, and research) (Unim et al., 2017). One of the challenges in the provision of genetic services is the effective coordination of the different components of a delivery model while guaranteeing that genomic applications with proven efficacy and effectiveness are actually delivered to populations. The T3 research phase addresses such issues by increasing the spread of knowledge about evidence-based interventions (dissemination research), integrating these interventions into existing programs and structures (implementation research), and promoting the adoption of these interventions by stakeholders (diffusion research) (Khoury et al., 2007).

Given current economic constraints (Saltman and Cahn, 2013), the integration of evidence-based interventions into existing genetic programs (i.e., healthcare programs providing a genetic test) is likely to be more effective than establishing stand-alone programs in terms of allocation of resources and service organization. The main components of a genetic program are target population, genetic counseling, genetic testing, diagnosis of carrier status and the healthcare pathway based on the carrier status. A careful consideration of these components and the application of implementation research are necessary to develop a framework that can support and sustain effective genetic programs and services.

### Research Question

The specific questions guiding this review are: (i) What are genetic service delivery models? (ii) Who delivers genetic services and where? (iii) Who pays for genetic services and how? (iv) How do providers get paid? (v) Where do providers and consumers get information? (vi) What is the government's role? (vii) What are the cultural, ethical and policy issues? (viii) What are alternative delivery models? (ix) What public policy changes do we need? (x) How is information about genetic services disseminated? (Washington State Department of Health, 2008).

Each genetic program is characterized by a specific genetic service delivery model which is defined by several factors such as: (i) practice setting and financial resources (public vs. private); (ii) service provider and patient access [geneticists vs. primary care physicians/other medical specialists (e.g., cardiologists, oncologists, neurologists, endocrinologists, and so on)]; (iii) policy regulation (national and local policies, guidelines, protocols, and position statements); (iv) laboratory practice standards (quality control standards, qualified personnel, etc.); and (v) information dissemination (methods of providing information about genetic services to patients and service providers). These factors, which are unique to each genetic service delivery model and depend on organizational aspects of the genetic service and on patient characteristics, will be considered in the present literature review. We aim to evaluate genetic services in order to identify and classify the existing genetic service delivery models for the provision of genetic testing in European and extra-European (Anglophone) countries (Canada, USA, Australia, or New Zealand).

## Methods

### Study Design

This systematic review of the literature was carried out within a European multicenter project “The Personalized pREvention of Chronic Diseases-PRECeDI (Marie Sklodowska-Curie Research and Innovation Staff Exchange 2014).”

### Systematic Review Protocol

The PRECeDI project is conducted through a multidimensional approach, which includes (i) a preliminary (non-systematic) literature search to identify and define the terms “genetic service” and “genetic service delivery models;” (ii) a systematic review of published literature on existing genetic service delivery models and selected country websites for policy documents; (iii) structured interviews with health experts on genetic service delivery models, policies governing the use of genomics medicine, and evaluation of genetic testing and related services in their respective countries; and (iv) a survey of European Public Health Association (EUPHA) members' knowledge and attitudes regarding the use of genomic applications in clinical practice. This review focuses on genetic service delivery models; thus, results of the policy review, the cross-sectional studies addressing European health experts and EUPHA members are not reported. Moreover, details of the preliminary non-systematic research can be found in the research protocol (Unim et al., 2017).

### Search Strategy, Participants, Interventions, and Comparators

The research was conducted according to the Preferred Reporting Items for Systematic Reviews and Meta-Analyses (PRISMA) Statement (Liberati et al., 2009). Two investigators independently searched five medical electronic resources (PubMed, Scopus, Web of Science, Google, and Google Scholar) using the following search terms: *genetic(s) services OR genetic(s) service provision OR genetic(s) service delivery OR genomic service delivery OR genetic(s) delivery models*. A preliminary non-systematic search and a manual review of references from relevant systematic reviews were also performed. The inclusion criteria were: (i) relevant articles and reports on pilot studies, best practices, and funded projects inherent to genetic service delivery; (ii) provision of all types of genetic tests by genetic specialist teams and healthcare professionals practicing in primary or secondary care; (iii) studies published in English and Italian between 2000 and 2015; and (iv) interventions carried out in European and extra-European (Anglophone) countries (the USA, Canada, Australia, and New Zealand). The extra-European countries were used for comparison purposes only. The exclusion criteria were: (i) studies reporting only on genetic counseling services; (ii) descriptive studies where pathways to care were not well defined; and (iii) studies not specifying the type of genetic test considered.

### Data Sources, Studies Sections and Data Extraction

An *ad-hoc* data extraction form was developed to collect relevant information from the included studies and is composed of three sections (Supplementary Material):

- General description of the study and the genetic service. This section collects general information about the study (i.e., authors, title of the study, country/region where the genetic service is implemented, etc.), the genetic service and its programs (i.e., practice setting, financing mechanism, type of healthcare system in the country, existence of national or regional policies on genetic services, etc.);
- Information on patients and pathways to care. This section investigates the characteristics of the target population of the genetic programs offered (i.e., gender, age, ethnicity) and pathways to care, as well as cost-effectiveness and efficacy of the genetic program;
- Genetic service evaluation. This section investigates the strengths and weaknesses of the genetic service and its programs in regard to cost-effectiveness and feasibility of the genetic programs, the genetic service capacity in terms of population and geographic area served, staff qualification, and laboratory standards.

### Data Analysis

Four members of the working group made an independent evaluation of each genetic service and the genetic programs offered using the data extraction form, followed by extensive group discussions. Any discrepancies in individual evaluations were resolved after discussion with the coordinators of the project.

For the different types of genetic testing considered in the review (i.e., prenatal, preimplantation, diagnostic, carrier, predictive, presymptomatic, newborn screening), the definitions of the National Institutes of Health (NIH) were adopted [The National Institutes of Health (NIH), 2018]. Throughout the review, a care pathway is defined as the patient flow through different professionals from the initial point of access to healthcare services to treatment of the genetic disorder and follow-up. The studies identified through the literature review were used for the classification of current genetic service delivery models.

## Results

### Study Selection and Characteristics

The preliminary literature search produced six records that were useful in defining and identifying genetic services and different models of genetic service delivery (Washington State Department of Health, 2008; Gu and Warren, 2009; Little et al., 2009; Metcalfe et al., 2009; Gu et al., 2011; Battista et al., 2012), although only Gu et al. (Gu and Warren, 2009; Gu et al., 2011) and Battista et al. (2012) classified their models. Thus, Battista et al. (2012) identified four types of genetic service delivery model according to the medical specialties of the healthcare professionals involved in service provision in each model: (i) multidisciplinary specialist clinics and coordinated services in rare genetic disorders led by geneticists; (ii) genetic services integrated with other medical specialties (e.g., oncogenetics, neurogenetics, cardiogenetics); (iii) genetic services integrated into primary care; and iv) genetic services provided in screening programs (i.e., prenatal and newborn screening). The classification provided by Gu et al. (Gu and Warren, 2009; Gu et al., 2011) focuses mainly on the patient pathway from the point of access to the genetic service to diagnosis and treatment of the genetic disorder: (i) The Patient-Doctor-Counselor Model; (ii) The Patient-Doctor-Lab Model; (iii) The Patient-Counselor-Lab Model; and (iv) The Patient-Lab (Commercial) Model (i.e., direct-to-consumer genetic testing).

**Figure 1:**
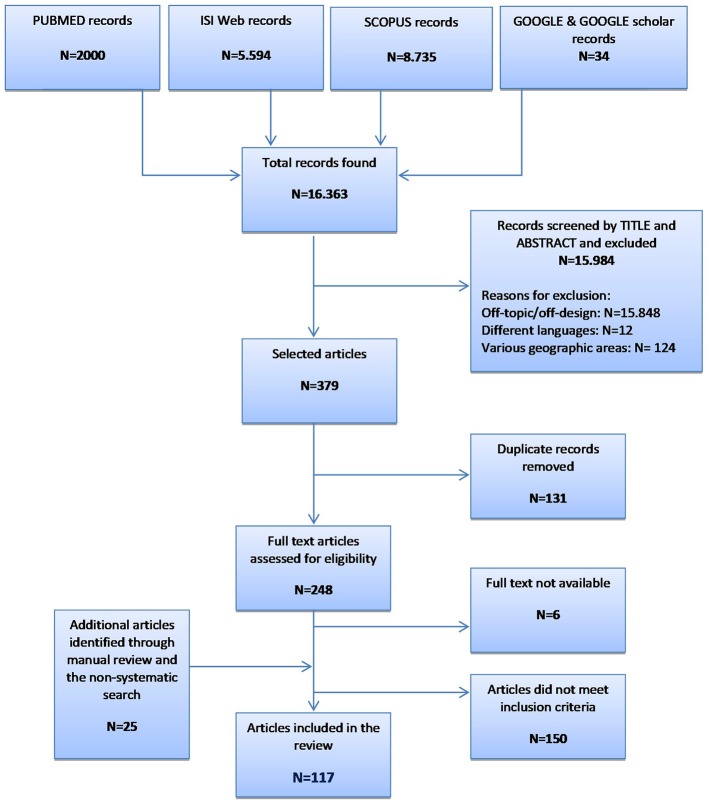
Flow diagram of the selection process.

Using five electronic resources, we retrieved more than 16,000 records (Figure 1). After evaluation of titles and abstracts, we excluded the majority for the following reasons: (i) off-topic/off-design; (ii) published in languages other than English or Italian (i.e., two in German, five in Japanese, three in Czech, one in Norwegian, and one in Danish); and (iii) performed in geographic areas not considered by the present study (i.e., Latin America, Asia, and Africa). Up to 150 articles did not meet the inclusion criteria on the description of the genetic programs (i.e., target population, genetic counseling, genetic testing, diagnosis of carrier status and the healthcare pathway based on the carrier status). Most articles focused on only one of these aspects, mainly genetic counseling, and were therefore excluded. The present systematic review consists of 117 records (Brain et al., 2000, 2002; Gray et al., 2000; Harper et al., 2000; Massie et al., 2000; Pichert and Stahel, 2000; Bach et al., 2001; Bickerstaff et al., 2001; Donnai and Elles, 2001; Ekstein and Katzenstein, 2001; Heath et al., 2001; Shepherd et al., 2001, 2003, 2014; Wonderling et al., 2001; Charron et al., 2002; Hartenbach et al., 2002; Lee et al., 2002; Lena-Russo et al., 2002; The Genetic Services Plan for Wisconsin, 2002; Anton-Culver et al., 2003; Barlow-Stewart et al., 2003; Campbell et al., 2003; Fry et al., 2003; Gason et al., 2003, 2005; Hopwood et al., 2003; Menkiszak et al., 2003; Rowland et al., 2003; Salbert, 2003; Comeau et al., 2004; Henriksson et al., 2004; Holloway et al., 2004; Kornreich et al., 2004; Basran et al., 2005; Calzolari and Baroncini, 2005; Epplein et al., 2005; Gozdzik et al., 2005; Hanley, 2005; Henry et al., 2005; Byck et al., 2006; Foretova et al., 2006; Gronwald et al., 2006; Mackay and Taylor, 2006; Puryear et al., 2006; Reis et al., 2006; Ricker et al., 2006; Therrell et al., 2006; Westwood et al., 2006; Windmill and Windmill, 2006; Young et al., 2006; Allen et al., 2007; Bennett et al., 2007, 2010; Berkenstadt et al., 2007; Brennan et al., 2007; Drury et al., 2007; Eeles et al., 2007; Gulzar et al., 2007; Mak et al., 2007; Morad et al., 2007; Southern et al., 2007; Srinivasa et al., 2007; Tozer and Lugton, 2007; Williams et al., 2007; Coffey et al., 2008; Eisinger, 2008; Kaye, 2008; Washington State Department of Health, 2008; Williamson and LeBlanc, 2008; Evans et al., 2009, 2012; Gu and Warren, 2009; Little et al., 2009; McCann et al., 2009; Metcalfe et al., 2009; Moeschler et al., 2009; Mulsow et al., 2009; Schofield et al., 2009, 2014; Smith et al., 2009; Streetly et al., 2009; Burton et al., 2010; Shields et al., 2010; Speechley and Nisker, 2010; Thuret et al., 2010; Watts et al., 2010; Aarden et al., 2011; Gu et al., 2011; Hoppe, 2011; Kaufmann et al., 2011; McGuire and Burke, 2011; Battista et al., 2012; Blumenfeld et al., 2012; Burton, 2012; Currier et al., 2012; Hamblion et al., 2012; Pohjola et al., 2012; Eble et al., 2013; Orlando et al., 2013, 2014; Pujol et al., 2013; Ramsden et al., 2013; Turcu et al., 2013; Amato et al., 2014; Bell et al., 2014, 2015; Kirk et al., 2014; Koeneman et al., 2014; Long and Goldblatt, 2014; Lucci et al., 2014; Mogayzel et al., 2014; Nesbitt et al., 2014; O'Brien et al., 2014; Plunkett et al., 2014; Vickery et al., 2014; Kirke et al., 2015; Slade et al., 2015) published from 2000 to 2015.

### Synthesized Findings

#### Genetic Services and Programs

##### General characteristics of the genetic programs

**Figure 2:**
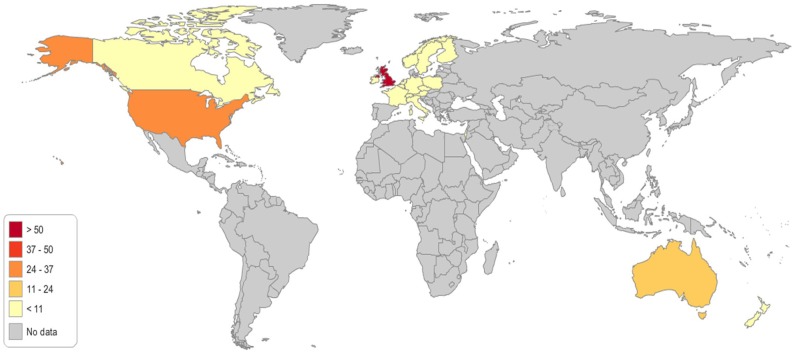
Geographical distribution of the genetic programs identified through the literature review.

A total of 148 genetic programs, implemented between 1960 and 2012, were identified. Thirteen records described more than one genetic program (Brain et al., 2000; Gray et al., 2000; Campbell et al., 2003; Fry et al., 2003; Hopwood et al., 2003; Holloway et al., 2004; Washington State Department of Health, 2008; Evans et al., 2009; Gu and Warren, 2009; Thuret et al., 2010; Aarden et al., 2011; Burton, 2012; Amato et al., 2014). Most of the programs were delivered in either the UK (59; 40%) (Brain et al., 2000, 2002; Gray et al., 2000; Harper et al., 2000; Bickerstaff et al., 2001; Donnai and Elles, 2001; Heath et al., 2001; Shepherd et al., 2001, 2003, 2014; Wonderling et al., 2001; Campbell et al., 2003; Fry et al., 2003; Hopwood et al., 2003; Holloway et al., 2004; Mackay and Taylor, 2006; Reis et al., 2006; Westwood et al., 2006; Young et al., 2006; Allen et al., 2007; Bennett et al., 2007, 2010; Brennan et al., 2007; Drury et al., 2007; Eeles et al., 2007; Gulzar et al., 2007; Mak et al., 2007; Southern et al., 2007; Srinivasa et al., 2007; Tozer and Lugton, 2007; Williams et al., 2007; Kaye, 2008; Evans et al., 2009, 2012; McCann et al., 2009; Streetly et al., 2009; Burton et al., 2010; Shields et al., 2010; Aarden et al., 2011; Hamblion et al., 2012; Ramsden et al., 2013; Turcu et al., 2013; Kirk et al., 2014; Nesbitt et al., 2014; Slade et al., 2015), the USA (35; 24%) (Bach et al., 2001; Ekstein and Katzenstein, 2001; Hartenbach et al., 2002; The Genetic Services Plan for Wisconsin, 2002; Anton-Culver et al., 2003; Salbert, 2003; Comeau et al., 2004; Kornreich et al., 2004; Epplein et al., 2005; Henry et al., 2005; Byck et al., 2006; Puryear et al., 2006; Ricker et al., 2006; Therrell et al., 2006; Windmill and Windmill, 2006; Coffey et al., 2008; Washington State Department of Health, 2008; Williamson and LeBlanc, 2008; Moeschler et al., 2009; Smith et al., 2009; Hoppe, 2011; McGuire and Burke, 2011; Blumenfeld et al., 2012; Currier et al., 2012; Eble et al., 2013; Orlando et al., 2013, 2014; Mogayzel et al., 2014) or Australia (16; 11%) (Massie et al., 2000; Ekstein and Katzenstein, 2001; Barlow-Stewart et al., 2003; Gason et al., 2003, 2005; Rowland et al., 2003; Metcalfe et al., 2009; Schofield et al., 2009, 2014; Watts et al., 2010; Bell et al., 2014; Long and Goldblatt, 2014; Vickery et al., 2014; Kirke et al., 2015) (Figure 2) and were available at national level (66; 45%), regional level (49; 33%) or only in urban areas (21; 14%) (Table 1). Nine programs were offered only locally (e.g., community health centers) while three programs served rural areas (Tozer and Lugton, 2007; Williamson and LeBlanc, 2008; McCann et al., 2009).

**Table 1:** General characteristics of the genetic services.

**General characteristics**	**References**
**GEOGRAPHICAL DISTRIBUTION**	
National level	Brain et al., 2000; Gray et al., 2000; Harper et al., 2000; Pichert and Stahel, 2000; Bach et al., 2001; Bickerstaff et al., 2001; Ekstein and Katzenstein, 2001; Heath et al., 2001; The Genetic Services Plan for Wisconsin, 2002; Anton-Culver et al., 2003; Menkiszak et al., 2003; Shepherd et al., 2003, 2014; Kornreich et al., 2004; Epplein et al., 2005; Gozdzik et al., 2005; Hanley, 2005; Foretova et al., 2006; Gronwald et al., 2006; Puryear et al., 2006; Therrell et al., 2006; Bennett et al., 2007; Berkenstadt et al., 2007; Southern et al., 2007; Eisinger, 2008; Kaye, 2008; Washington State Department of Health, 2008; Evans et al., 2009, 2012; Gu and Warren, 2009; Little et al., 2009; Metcalfe et al., 2009; Streetly et al., 2009; Burton et al., 2010; Shields et al., 2010; Speechley and Nisker, 2010; Thuret et al., 2010; Aarden et al., 2011; Gu et al., 2011; McGuire and Burke, 2011; Burton, 2012; Hamblion et al., 2012; Pohjola et al., 2012; Pujol et al., 2013; Turcu et al., 2013; Kirk et al., 2014; Mogayzel et al., 2014; O'Brien et al., 2014; Slade et al., 2015
Regional level	Brain et al., 2000, 2002; Gray et al., 2000; Massie et al., 2000; Donnai and Elles, 2001; Wonderling et al., 2001; Hartenbach et al., 2002; Lee et al., 2002; The Genetic Services Plan for Wisconsin, 2002; Campbell et al., 2003; Fry et al., 2003; Rowland et al., 2003; Salbert, 2003; Comeau et al., 2004; Henriksson et al., 2004; Holloway et al., 2004; Basran et al., 2005; Calzolari and Baroncini, 2005; Henry et al., 2005; Byck et al., 2006; Mackay and Taylor, 2006; Reis et al., 2006; Windmill and Windmill, 2006; Brennan et al., 2007; Gulzar et al., 2007; Mak et al., 2007; Williams et al., 2007; Coffey et al., 2008; Moeschler et al., 2009; Schofield et al., 2009, 2014; Bennett et al., 2010; Hoppe, 2011; Blumenfeld et al., 2012; Currier et al., 2012; Orlando et al., 2013; Amato et al., 2014; Long and Goldblatt, 2014; Lucci et al., 2014; Vickery et al., 2014; Bell et al., 2015; Kirke et al., 2015
Urban areas	Charron et al., 2002; Lena-Russo et al., 2002; Barlow-Stewart et al., 2003; Gason et al., 2003, 2005; Hopwood et al., 2003; Ricker et al., 2006; Morad et al., 2007; Mulsow et al., 2009; Smith et al., 2009; Kaufmann et al., 2011; Eble et al., 2013; Ramsden et al., 2013; Koeneman et al., 2014; Nesbitt et al., 2014
Local level	Shepherd et al., 2001; Allen et al., 2007; Drury et al., 2007; Eeles et al., 2007; Srinivasa et al., 2007; Thuret et al., 2010; Orlando et al., 2014; Bell et al., 2015
**LEVEL OF IMPLEMENTATION**	
Integrated into healthcare systems	Brain et al., 2000, 2002; Gray et al., 2000; Harper et al., 2000; Massie et al., 2000; Bach et al., 2001; Bickerstaff et al., 2001; Donnai and Elles, 2001; Ekstein and Katzenstein, 2001; Heath et al., 2001; Hartenbach et al., 2002; Lee et al., 2002; Lena-Russo et al., 2002; The Genetic Services Plan for Wisconsin, 2002; Campbell et al., 2003; Fry et al., 2003; Hopwood et al., 2003; Menkiszak et al., 2003; Rowland et al., 2003; Salbert, 2003; Shepherd et al., 2003, 2014; Comeau et al., 2004; Henriksson et al., 2004; Holloway et al., 2004; Kornreich et al., 2004; Basran et al., 2005; Calzolari and Baroncini, 2005; Epplein et al., 2005; Gason et al., 2005; Gozdzik et al., 2005; Hanley, 2005; Henry et al., 2005; Byck et al., 2006; Foretova et al., 2006; Mackay and Taylor, 2006; Puryear et al., 2006; Reis et al., 2006; Therrell et al., 2006; Windmill and Windmill, 2006; Berkenstadt et al., 2007; Mak et al., 2007; Morad et al., 2007; Southern et al., 2007; Williams et al., 2007; Coffey et al., 2008; Eisinger, 2008; Washington State Department of Health, 2008; Evans et al., 2009; Gu and Warren, 2009; Little et al., 2009; Metcalfe et al., 2009; Streetly et al., 2009; Burton et al., 2010; Shields et al., 2010; Speechley and Nisker, 2010; Thuret et al., 2010; Watts et al., 2010; Aarden et al., 2011; Hoppe, 2011; Kaufmann et al., 2011; Blumenfeld et al., 2012; Burton, 2012; Currier et al., 2012; Hamblion et al., 2012; Eble et al., 2013; Turcu et al., 2013; Amato et al., 2014; Long and Goldblatt, 2014; Lucci et al., 2014; Mogayzel et al., 2014; Nesbitt et al., 2014; O'Brien et al., 2014; Plunkett et al., 2014; Schofield et al., 2014; Bell et al., 2015; Slade et al., 2015
Pilot services	Brain et al., 2000, 2002; Gray et al., 2000; Pichert and Stahel, 2000; Charron et al., 2002; Anton-Culver et al., 2003; Barlow-Stewart et al., 2003; Campbell et al., 2003; Fry et al., 2003; Gason et al., 2003; Holloway et al., 2004; Gronwald et al., 2006; Mackay and Taylor, 2006; Ricker et al., 2006; Westwood et al., 2006; Young et al., 2006; Allen et al., 2007; Bennett et al., 2007, 2010; Brennan et al., 2007; Drury et al., 2007; Eeles et al., 2007; Gulzar et al., 2007; Southern et al., 2007; Srinivasa et al., 2007; Tozer and Lugton, 2007; Williamson and LeBlanc, 2008; Evans et al., 2009, 2012; McCann et al., 2009; Moeschler et al., 2009; Mulsow et al., 2009; Schofield et al., 2009; Smith et al., 2009; Thuret et al., 2010; Gu et al., 2011; Burton, 2012; Pohjola et al., 2012; Orlando et al., 2013, 2014; Pujol et al., 2013; Ramsden et al., 2013; Kirk et al., 2014; Koeneman et al., 2014; Bell et al., 2015; Kirke et al., 2015
DTC services	Kaye, 2008; Washington State Department of Health, 2008; Gu and Warren, 2009; Gu et al., 2011; McGuire and Burke, 2011
**INFORMATION DISSEMINATION TO HCPs AND ICTs**	
Professional boards	Donnai and Elles, 2001; The Genetic Services Plan for Wisconsin, 2002; Campbell et al., 2003; Fry et al., 2003; Hopwood et al., 2003; Salbert, 2003; Holloway et al., 2004; Hanley, 2005; Henry et al., 2005; Puryear et al., 2006; Srinivasa et al., 2007; Tozer and Lugton, 2007; Washington State Department of Health, 2008; Williamson and LeBlanc, 2008; Little et al., 2009; Metcalfe et al., 2009; Eble et al., 2013
Conferences, meetings, workshops	Anton-Culver et al., 2003; Salbert, 2003; Comeau et al., 2004; Calzolari and Baroncini, 2005; Mackay and Taylor, 2006; Puryear et al., 2006; Ricker et al., 2006; Bennett et al., 2007, 2010; Brennan et al., 2007; Srinivasa et al., 2007; Tozer and Lugton, 2007; Moeschler et al., 2009; Smith et al., 2009; Shields et al., 2010; Thuret et al., 2010; Mogayzel et al., 2014
Scientific journals	Campbell et al., 2003; Fry et al., 2003; Hopwood et al., 2003; Holloway et al., 2004; Reis et al., 2006; Ricker et al., 2006; Young et al., 2006; Eble et al., 2013
No information dissemination to patients	Massie et al., 2000; Bach et al., 2001; Heath et al., 2001; Shepherd et al., 2001; Wonderling et al., 2001; Brain et al., 2002; Hartenbach et al., 2002; Lee et al., 2002; Basran et al., 2005; Foretova et al., 2006; Bennett et al., 2007; Southern et al., 2007; Eisinger, 2008; Evans et al., 2009; McCann et al., 2009; Moeschler et al., 2009; Streetly et al., 2009; Aarden et al., 2011; Gu et al., 2011; Kaufmann et al., 2011; Burton, 2012; Hamblion et al., 2012; Turcu et al., 2013; Kirk et al., 2014; Bell et al., 2015; Kirke et al., 2015; Slade et al., 2015
ICTs for medical records	Brain et al., 2000; Gray et al., 2000; Wonderling et al., 2001; Lee et al., 2002; Anton-Culver et al., 2003; Rowland et al., 2003; Salbert, 2003; Epplein et al., 2005; Hanley, 2005; Byck et al., 2006; Reis et al., 2006; Therrell et al., 2006; Young et al., 2006; Allen et al., 2007; Drury et al., 2007; Eeles et al., 2007; Mak et al., 2007; Tozer and Lugton, 2007; Williams et al., 2007; Washington State Department of Health, 2008; Williamson and LeBlanc, 2008; Moeschler et al., 2009; Mulsow et al., 2009; Schofield et al., 2009, 2014; Smith et al., 2009; Bennett et al., 2010; Shields et al., 2010; Speechley and Nisker, 2010; Gu et al., 2011; Hoppe, 2011; Evans et al., 2012; Orlando et al., 2013, 2014; Bell et al., 2014; Koeneman et al., 2014; Lucci et al., 2014; Mogayzel et al., 2014; Vickery et al., 2014
ICTs for internet-based services	The Genetic Services Plan for Wisconsin, 2002; Anton-Culver et al., 2003; Hopwood et al., 2003; Henriksson et al., 2004; Epplein et al., 2005; Mackay and Taylor, 2006; Bennett et al., 2007; Tozer and Lugton, 2007; Kaye, 2008; Washington State Department of Health, 2008; Gu and Warren, 2009; McCann et al., 2009; Gu et al., 2011; McGuire and Burke, 2011; Blumenfeld et al., 2012; Evans et al., 2012; Orlando et al., 2013; Shepherd et al., 2014; Kirke et al., 2015; Slade et al., 2015
**TRAINING IN GENETICS**	
Training resources	Pichert and Stahel, 2000; Donnai and Elles, 2001; Wonderling et al., 2001; The Genetic Services Plan for Wisconsin, 2002; Hopwood et al., 2003; Salbert, 2003; Shepherd et al., 2003, 2014; Henriksson et al., 2004; Calzolari and Baroncini, 2005; Epplein et al., 2005; Gozdzik et al., 2005; Henry et al., 2005; Byck et al., 2006; Puryear et al., 2006; Reis et al., 2006; Ricker et al., 2006; Allen et al., 2007; Bennett et al., 2007; Brennan et al., 2007; Drury et al., 2007; Eeles et al., 2007; Gulzar et al., 2007; Srinivasa et al., 2007; Eisinger, 2008; Washington State Department of Health, 2008; Williamson and LeBlanc, 2008; Little et al., 2009; Metcalfe et al., 2009; Moeschler et al., 2009; Streetly et al., 2009; Shields et al., 2010; Thuret et al., 2010; Watts et al., 2010; Blumenfeld et al., 2012; Eble et al., 2013; Orlando et al., 2013; Kirk et al., 2014; Mogayzel et al., 2014
Physicians and nurses	Pichert and Stahel, 2000; Donnai and Elles, 2001; Wonderling et al., 2001; The Genetic Services Plan for Wisconsin, 2002; Anton-Culver et al., 2003; Campbell et al., 2003; Fry et al., 2003; Hopwood et al., 2003; Salbert, 2003; Shepherd et al., 2003; Comeau et al., 2004; Holloway et al., 2004; Calzolari and Baroncini, 2005; Epplein et al., 2005; Gozdzik et al., 2005; Henry et al., 2005; Byck et al., 2006; Mackay and Taylor, 2006; Puryear et al., 2006; Ricker et al., 2006; Therrell et al., 2006; Young et al., 2006; Bennett et al., 2007, 2010; Brennan et al., 2007; Drury et al., 2007; Eeles et al., 2007; Srinivasa et al., 2007; Tozer and Lugton, 2007; Eisinger, 2008; Washington State Department of Health, 2008; Little et al., 2009; Metcalfe et al., 2009; Burton et al., 2010; Shields et al., 2010; Thuret et al., 2010; Watts et al., 2010; Blumenfeld et al., 2012; Burton, 2012; Eble et al., 2013; Orlando et al., 2013; Kirk et al., 2014; Mogayzel et al., 2014; Vickery et al., 2014; Bell et al., 2015
Assistants, genetic counselors, biologists, social workers, midwives	Pichert and Stahel, 2000; Donnai and Elles, 2001; Wonderling et al., 2001; The Genetic Services Plan for Wisconsin, 2002; Anton-Culver et al., 2003; Campbell et al., 2003; Fry et al., 2003; Hopwood et al., 2003; Salbert, 2003; Shepherd et al., 2003, 2014; Comeau et al., 2004; Henriksson et al., 2004; Holloway et al., 2004; Calzolari and Baroncini, 2005; Epplein et al., 2005; Gozdzik et al., 2005; Henry et al., 2005; Byck et al., 2006; Mackay and Taylor, 2006; Puryear et al., 2006; Reis et al., 2006; Ricker et al., 2006; Therrell et al., 2006; Young et al., 2006; Allen et al., 2007; Brennan et al., 2007; Drury et al., 2007; Eeles et al., 2007; Gulzar et al., 2007; Morad et al., 2007; Srinivasa et al., 2007; Eisinger, 2008; Washington State Department of Health, 2008; Williamson and LeBlanc, 2008; Little et al., 2009; Metcalfe et al., 2009; Moeschler et al., 2009; Streetly et al., 2009; Bennett et al., 2010; Shields et al., 2010; Thuret et al., 2010; Watts et al., 2010; Blumenfeld et al., 2012; Burton, 2012; Eble et al., 2013; Orlando et al., 2013; Bell et al., 2014; Kirk et al., 2014; Mogayzel et al., 2014; Vickery et al., 2014
**GENETIC LABORATORIES**	
National and regional regulations	Pichert and Stahel, 2000; Bach et al., 2001; Bickerstaff et al., 2001; Donnai and Elles, 2001; Ekstein and Katzenstein, 2001; Heath et al., 2001; Charron et al., 2002; Barlow-Stewart et al., 2003; Kornreich et al., 2004; Calzolari and Baroncini, 2005; Byck et al., 2006; Foretova et al., 2006; Therrell et al., 2006; Southern et al., 2007; Eisinger, 2008; Washington State Department of Health, 2008; Little et al., 2009; Streetly et al., 2009; Shields et al., 2010; Kirke et al., 2015
Affiliation with local genetic services	Harper et al., 2000; Heath et al., 2001; Charron et al., 2002; Hopwood et al., 2003; Shepherd et al., 2003; Epplein et al., 2005; Byck et al., 2006; Puryear et al., 2006; Morad et al., 2007; Southern et al., 2007; Shields et al., 2010; Thuret et al., 2010; Aarden et al., 2011; Hoppe, 2011; Kaufmann et al., 2011; O'Brien et al., 2014
Affiliation with regional genetic services	Harper et al., 2000; Pichert and Stahel, 2000; Bickerstaff et al., 2001; Donnai and Elles, 2001; Heath et al., 2001; Wonderling et al., 2001; Rowland et al., 2003; Shepherd et al., 2003; Calzolari and Baroncini, 2005; Byck et al., 2006; Therrell et al., 2006; Mak et al., 2007; Southern et al., 2007; Washington State Department of Health, 2008; Schofield et al., 2009, 2014; Shields et al., 2010; Thuret et al., 2010; Aarden et al., 2011; Gu et al., 2011; Hoppe, 2011; Ramsden et al., 2013; Turcu et al., 2013; Bell et al., 2014; O'Brien et al., 2014
Affiliation with academic centers	Shepherd et al., 2003; Kornreich et al., 2004; Basran et al., 2005; Calzolari and Baroncini, 2005; Byck et al., 2006; Foretova et al., 2006; Puryear et al., 2006; Therrell et al., 2006; Southern et al., 2007; Washington State Department of Health, 2008; Metcalfe et al., 2009; Schofield et al., 2009, 2014; Shields et al., 2010; Thuret et al., 2010; Aarden et al., 2011; Gu et al., 2011; Hoppe, 2011; Kaufmann et al., 2011; McGuire and Burke, 2011; Blumenfeld et al., 2012; O'Brien et al., 2014
Private sector	Lee et al., 2002; Kornreich et al., 2004; Foretova et al., 2006; Puryear et al., 2006; Therrell et al., 2006; Washington State Department of Health, 2008; Gu and Warren, 2009; Little et al., 2009; Metcalfe et al., 2009; Schofield et al., 2009, 2014; Speechley and Nisker, 2010; Gu et al., 2011; Bell et al., 2014

*DTC, direct-to-consumer; HCPs, healthcare providers; ICTs, Information and communications technologies*.

A national plan within a PHG framework was reported only in the Italian setting (3 out of 6 programs) (Calzolari and Baroncini, 2005; Southern et al., 2007; Lucci et al., 2014), while regional or national guidelines on genetic services were reported for most genetic programs available worldwide. The programs were predominantly offered in the public sector, of which eight were in the academic sector (Hartenbach et al., 2002; Lee et al., 2002; Barlow-Stewart et al., 2003; Henriksson et al., 2004; Gozdzik et al., 2005; Brennan et al., 2007; Coffey et al., 2008; Mogayzel et al., 2014). The vast majority of programs (90) were publicly funded, with only eight using private funds (Pichert and Stahel, 2000; Hartenbach et al., 2002; Kaye, 2008; Washington State Department of Health, 2008; Gu and Warren, 2009; Gu et al., 2011; McGuire and Burke, 2011); most of the latter were direct-to-consumer (DTC) genetic services.

Ninety-six (64%) genetic programs were integrated into healthcare systems, while 48 (32%) were pilot programs and five (3%) were DTC services (Table 1). Two studies described both pilot programs and integrated services (Mackay and Taylor, 2006; Southern et al., 2007).

Genetic tests, most commonly BRCA1/2 (59, 40%), Lynch syndrome (23; 16%), and the newborn screening panel (18; 12%) (Table 2), were offered in 145/148 genetic programs. Other genetic tests offered were for various disorders (e.g., hemoglobinopathies, chromosomal abnormalities, endocrine disorders, inherited cardiovascular conditions, etc.) and oncological conditions (e.g., colorectal cancer, bowel cancer, etc.). Regarding geographical distribution, BRCA1/2 testing was mostly provided in the UK (29; 49%) and the USA (11; 19%), Lynch syndrome testing in the UK (6; 26%), and the newborn screening panel in the USA (9; 50%).

**Table 2:** Genetic testing and screening offered to individuals affected by or at risk of various genetic disorders.

**Genetic screening and testing**	**N programs**	**Country**	**References**
BRCA 1/2	59	UK (29); USA (11); other (1–3)	Brain et al., 2000, 2002; Gray et al., 2000; Pichert and Stahel, 2000; Donnai and Elles, 2001; Wonderling et al., 2001; Hartenbach et al., 2002; Lee et al., 2002; Anton-Culver et al., 2003; Campbell et al., 2003; Fry et al., 2003; Hopwood et al., 2003; Menkiszak et al., 2003; Rowland et al., 2003; Henriksson et al., 2004; Holloway et al., 2004; Calzolari and Baroncini, 2005; Epplein et al., 2005; Foretova et al., 2006; Gronwald et al., 2006; Mackay and Taylor, 2006; Reis et al., 2006; Ricker et al., 2006; Westwood et al., 2006; Young et al., 2006; Allen et al., 2007; Bennett et al., 2007; Brennan et al., 2007; Drury et al., 2007; Eeles et al., 2007; Gulzar et al., 2007; Srinivasa et al., 2007; Tozer and Lugton, 2007; Eisinger, 2008; Washington State Department of Health, 2008; Williamson and LeBlanc, 2008; Evans et al., 2009, 2012; Little et al., 2009; Mulsow et al., 2009; Smith et al., 2009; Speechley and Nisker, 2010; Eble et al., 2013; Orlando et al., 2013, 2014; Pujol et al., 2013; Koeneman et al., 2014; Slade et al., 2015
Lynch syndrome	23	UK (6); other (1–3)	Pichert and Stahel, 2000; Wonderling et al., 2001; Hopwood et al., 2003; Henriksson et al., 2004; Epplein et al., 2005; Bennett et al., 2007; Mak et al., 2007; Williams et al., 2007; Eisinger, 2008; Schofield et al., 2009, 2014; Pujol et al., 2013; Koeneman et al., 2014; Orlando et al., 2014; Plunkett et al., 2014
Disorders included in the Newborn screening panel	18	USA (9); other (1–3)	Massie et al., 2000; The Genetic Services Plan for Wisconsin, 2002; Salbert, 2003; Comeau et al., 2004; Basran et al., 2005; Calzolari and Baroncini, 2005; Hanley, 2005; Henry et al., 2005; Byck et al., 2006; Puryear et al., 2006; Therrell et al., 2006; Washington State Department of Health, 2008; Little et al., 2009; Metcalfe et al., 2009; Streetly et al., 2009; Thuret et al., 2010; Mogayzel et al., 2014
Cystic Fibrosis	17	USA (7); Australia (5); UK (4); other (1–2)	Massie et al., 2000; Bickerstaff et al., 2001; Donnai and Elles, 2001; Ekstein and Katzenstein, 2001; Barlow-Stewart et al., 2003; Kornreich et al., 2004; Gozdzik et al., 2005; Byck et al., 2006; Drury et al., 2007; Southern et al., 2007; Washington State Department of Health, 2008; Metcalfe et al., 2009; Speechley and Nisker, 2010; Blumenfeld et al., 2012; Currier et al., 2012; Long and Goldblatt, 2014; Mogayzel et al., 2014
Hemoglobinopathies (alfa- and beta-thalassemia, HbS, HbC)	15	USA (3); other (1–2)	Bickerstaff et al., 2001; Lena-Russo et al., 2002; Basran et al., 2005; Washington State Department of Health, 2008; Metcalfe et al., 2009; Streetly et al., 2009; Speechley and Nisker, 2010; Thuret et al., 2010; Hoppe, 2011; Kaufmann et al., 2011; Currier et al., 2012; Amato et al., 2014; Long and Goldblatt, 2014
Familial hypercholesterolemia	11	Australia (5); UK (4); other (1)	Heath et al., 2001; Watts et al., 2010; Aarden et al., 2011; Burton, 2012; Bell et al., 2014, 2015; Kirk et al., 2014; Vickery et al., 2014; Kirke et al., 2015
Chromosomal abnormalities (trisomy 21, 18, and 13, 22q11 deletions, translocations, fragile X syndrome)	10	USA (5); Other (1–2)	Bickerstaff et al., 2001; Salbert, 2003; Byck et al., 2006; Washington State Department of Health, 2008; Little et al., 2009; Metcalfe et al., 2009; Speechley and Nisker, 2010; Currier et al., 2012; Eble et al., 2013; Long and Goldblatt, 2014
Tay-Sachs	8	Australia (4); USA (4); Other (1–2)	Bach et al., 2001; Bickerstaff et al., 2001; Ekstein and Katzenstein, 2001; The Genetic Services Plan for Wisconsin, 2002; Barlow-Stewart et al., 2003; Gason et al., 2003, 2005; Washington State Department of Health, 2008
Colorectal cancer	7	USA (3); other (1–2)	Anton-Culver et al., 2003; Brennan et al., 2007; Gulzar et al., 2007; Little et al., 2009; Eble et al., 2013; Orlando et al., 2013; Plunkett et al., 2014
Diabetes 1 and 2 genetic testing, MODY	7	UK (4); USA (3)	Shepherd et al., 2001, 2003, 2014; Washington State Department of Health, 2008; Shields et al., 2010; Burton, 2012; Orlando et al., 2013
Hereditary cancer syndromes (von Hippel-Linda, neurofibromatosis, Wilms tumor, Li Fraumeni, Cowden, etc.)	6	USA (4); other (1)	Wonderling et al., 2001; Henriksson et al., 2004; Epplein et al., 2005; Burton, 2012; Orlando et al., 2013, 2014
Adult onset diseases (Alzheimer, Huntington)	6	UK (4); other (1)	Harper et al., 2000; Bickerstaff et al., 2001; Donnai and Elles, 2001; Drury et al., 2007; Williamson and LeBlanc, 2008; Speechley and Nisker, 2010
Inherited cardiovascular conditions (arrhythmias, cardiomyopathies, inherited congenital heart disease, familial hyperlipidemia, etc.)	6	USA (3); other (1–2)	Charron et al., 2002; McCann et al., 2009; Burton et al., 2010; Burton, 2012; Eble et al., 2013; Kirk et al., 2014
Various disorders (thrombophilia, bowel cancer, cervical cancer, endometrial cancer, ovarian cancer, hereditary melanoma, hearing loss, developmental disabilities, surfactant dysfunction, mitochondrial diseases, carrier screening for genetic disorders in Ashkenazi Jews, etc.)	35	various settings (1–5)	Bickerstaff et al., 2001; Donnai and Elles, 2001; Ekstein and Katzenstein, 2001; Wonderling et al., 2001; The Genetic Services Plan for Wisconsin, 2002; Rowland et al., 2003; Salbert, 2003; Henriksson et al., 2004; Calzolari and Baroncini, 2005; Epplein et al., 2005; Henry et al., 2005; Windmill and Windmill, 2006; Berkenstadt et al., 2007; Brennan et al., 2007; Drury et al., 2007; Coffey et al., 2008; Kaye, 2008; Washington State Department of Health, 2008; Williamson and LeBlanc, 2008; Gu and Warren, 2009; Little et al., 2009; Metcalfe et al., 2009; Moeschler et al., 2009; Bennett et al., 2010; Gu et al., 2011; McGuire and Burke, 2011; Pohjola et al., 2012; Eble et al., 2013; Ramsden et al., 2013; Turcu et al., 2013; Lucci et al., 2014; Nesbitt et al., 2014; O'Brien et al., 2014

Three programs, which were part of a project described in three distinct studies (Brain et al., 2000, 2002; Gray et al., 2000), did not offer a genetic test. Each of these studies compared two programs, the standard service provided by breast surgeons vs. the service provided by a specialist genetics team in a newly established multidisciplinary genetics clinic. The trial group underwent risk assessment at the multidisciplinary genetics clinic and BRCA1/2 genetic testing was offered to women at high risk. The control group received only the standard program without genetic testing.

##### Information dissemination, communication technologies and training activities

Community healthcare providers (HCPs) were mainly informed about genetic services through professional boards; conferences, meetings and workshops; or scientific journals (Table 1). Other sources of information were websites, the genetic service staff, local genetic organizations or departments, and genetic networks using various advertisement materials (e.g., brochures, posters, letters, or emails to HCPs).

Various communication channels were also used to inform patients about the availability of genetic services, largely comprising HCPs, service websites, and the media (e.g., advertisements on radio and TV, and in magazines). Other means reported in some studies were distribution of educational materials (e.g., booklets, multilingual CDs, brochures, or pamphlets) by patient support organizations or genetic services; public education courses (e.g., via schools, prenatal courses); and community events organized by local organizations. However, in 35 genetic programs, information dissemination to patients was not reported by any means (Table 1).

Information and communications technologies (ICTs; e.g., cellular phones, computer, satellite systems) and associated services were used either alone or in combination to organize medical records (Table 1); for videoconferencing (Ricker et al., 2006; Moeschler et al., 2009); for distance learning (Campbell et al., 2003; Fry et al., 2003; Gason et al., 2003; Holloway et al., 2004; Puryear et al., 2006); and for various internet-based services including risk-assessment programs, telemedicine, and appointment scheduling programs. Genetic service providers also used ICTs to communicate with patients and community HCPs.

Genetic services offered their employees training in genetic medicine consisting of continuing education programs; seminars, conferences and workshops; provision of educational materials; interactive computer programs; referral guidelines; and staff supervision by geneticists or genetic counselors (Table 1). The training activities mostly addressed physicians and nurses, but also physicians' assistants, genetic counselors, biologists, social workers and midwives.

##### Staff qualifications

Several studies reported that physicians from various specialties had a specific background in medical genetics. Medical geneticists and other medical specialists (e.g., obstetrician-gynecologists, oncologists, cardiologists, endocrinologists, etc.) with genetics knowledge were more common than general practitioners (GPs). Among non-medical HCPs, genetic counselors (73/148 programs), laboratory staff (73/148 programs) and nurses (49/148 programs) also had a genetics background.

##### Characteristics of the laboratories

In 26 programs, the quality of the laboratories involved corresponded to the standards set out in regional or national regulations (Table 1). These programs were largely in the USA (12 programs) (Bach et al., 2001; Ekstein and Katzenstein, 2001; Kornreich et al., 2004; Byck et al., 2006; Washington State Department of Health, 2008; Streetly et al., 2009) and UK (6 programs) (Bickerstaff et al., 2001; Donnai and Elles, 2001; Heath et al., 2001; Southern et al., 2007; Streetly et al., 2009; Shields et al., 2010). Most of these laboratories were part of integrated services and operated in the public sector. Only one laboratory was part of a DTC service; it operated in the USA as a virtual clinic (Washington State Department of Health, 2008). The laboratories were affiliated with local genetic services; regional genetic services; academic centers; and some also operated in the private sector.

#### Patient Flow Through Clinical Genetics Care Pathways

##### General characteristics of the patients

Patients of both genders across a wide age range (1–62 years) used the genetic programs identified. Females were the only users in 31 programs, mainly for BRCA1/2 testing (Table 3). Pregnant women and couples were the targets of prenatal and preimplantation screening programs. Different programs were also offered to the pediatric population for disorders included in the newborn screening panel (cystic fibrosis -CF, phenylketonuria, galactosemia, hearing loss, etc.) (Table 3), Sotos syndrome (Pohjola et al., 2012), hereditary retinal diseases (Henriksson et al., 2004; Morad et al., 2007; Hamblion et al., 2012), developmental disabilities (Donnai and Elles, 2001; Moeschler et al., 2009) and more. Patient ethnicity was reported in 44 genetic programs and comprised all ethnic groups/races (e.g., Caucasians, Ashkenazi Jews, Hispanic, African-Americans, Asians) (Table 3).

**Table 3:** Patient flow through clinical genetics care pathways.

**Characteristics**	**References**
Female users (BRCA1/2 testing)	Brain et al., 2000, 2002; Hartenbach et al., 2002; Lee et al., 2002; Fry et al., 2003; Menkiszak et al., 2003; Holloway et al., 2004; Gronwald et al., 2006; Reis et al., 2006; Young et al., 2006; Allen et al., 2007; Mulsow et al., 2009; Smith et al., 2009; Evans et al., 2012; Koeneman et al., 2014
Newborn screening panel	Massie et al., 2000; The Genetic Services Plan for Wisconsin, 2002; Comeau et al., 2004; Basran et al., 2005; Calzolari and Baroncini, 2005; Hanley, 2005; Henry et al., 2005; Byck et al., 2006; Puryear et al., 2006; Therrell et al., 2006; Windmill and Windmill, 2006; Southern et al., 2007; Washington State Department of Health, 2008; Little et al., 2009; Metcalfe et al., 2009; Streetly et al., 2009; Thuret et al., 2010; Hoppe, 2011; Kaufmann et al., 2011; Mogayzel et al., 2014
Preimplantation screening	Massie et al., 2000; Berkenstadt et al., 2007; Little et al., 2009; McCann et al., 2009; Metcalfe et al., 2009; Speechley and Nisker, 2010; Kaufmann et al., 2011; Currier et al., 2012; Long and Goldblatt, 2014; Nesbitt et al., 2014
Ethnic groups/races	Bach et al., 2001; Ekstein and Katzenstein, 2001; Wonderling et al., 2001; Lee et al., 2002; Lena-Russo et al., 2002; Anton-Culver et al., 2003; Barlow-Stewart et al., 2003; Kornreich et al., 2004; Basran et al., 2005; Gason et al., 2005; Byck et al., 2006; Ricker et al., 2006; Westwood et al., 2006; Allen et al., 2007; Bennett et al., 2007, 2010; Eeles et al., 2007; Gulzar et al., 2007; Srinivasa et al., 2007; Coffey et al., 2008; Washington State Department of Health, 2008; Metcalfe et al., 2009; Smith et al., 2009; Streetly et al., 2009; Shields et al., 2010; Thuret et al., 2010; Kaufmann et al., 2011; Currier et al., 2012; Evans et al., 2012; Hamblion et al., 2012; Eble et al., 2013; Orlando et al., 2013, 2014; Amato et al., 2014; Lucci et al., 2014; Plunkett et al., 2014
**ACCESS TO GENETIC SERVICES**	
Direct access	Pichert and Stahel, 2000; Bach et al., 2001; Bickerstaff et al., 2001; Charron et al., 2002; Hartenbach et al., 2002; Anton-Culver et al., 2003; Campbell et al., 2003; Fry et al., 2003; Hopwood et al., 2003; Shepherd et al., 2003; Henriksson et al., 2004; Holloway et al., 2004; Epplein et al., 2005; Byck et al., 2006; Foretova et al., 2006; Gronwald et al., 2006; Puryear et al., 2006; Berkenstadt et al., 2007; Eeles et al., 2007; Gulzar et al., 2007; Morad et al., 2007; Tozer and Lugton, 2007; Coffey et al., 2008; Kaye, 2008; Washington State Department of Health, 2008; Williamson and LeBlanc, 2008; Gu and Warren, 2009; Little et al., 2009; Metcalfe et al., 2009; Mulsow et al., 2009; Smith et al., 2009; Gu et al., 2011; McGuire and Burke, 2011; Burton, 2012; Pujol et al., 2013; Ramsden et al., 2013; Amato et al., 2014; Nesbitt et al., 2014
Direct access to BRCA1/2 testing	Pichert and Stahel, 2000; Hartenbach et al., 2002; Anton-Culver et al., 2003; Campbell et al., 2003; Fry et al., 2003; Hopwood et al., 2003; Henriksson et al., 2004; Holloway et al., 2004; Epplein et al., 2005; Foretova et al., 2006; Gronwald et al., 2006; Westwood et al., 2006; Allen et al., 2007; Eeles et al., 2007; Gulzar et al., 2007; Tozer and Lugton, 2007; Williams et al., 2007; Washington State Department of Health, 2008; Williamson and LeBlanc, 2008; Little et al., 2009; McCann et al., 2009; Mulsow et al., 2009; Smith et al., 2009; Streetly et al., 2009; Pujol et al., 2013; Slade et al., 2015
Mediated by medical specialists and GPs	Brain et al., 2000, 2002; Gray et al., 2000; Pichert and Stahel, 2000; Bickerstaff et al., 2001; Donnai and Elles, 2001; Heath et al., 2001; Wonderling et al., 2001; Lee et al., 2002; Campbell et al., 2003; Fry et al., 2003; Hopwood et al., 2003; Rowland et al., 2003; Salbert, 2003; Henriksson et al., 2004; Holloway et al., 2004; Epplein et al., 2005; Henry et al., 2005; Byck et al., 2006; Mackay and Taylor, 2006; Reis et al., 2006; Ricker et al., 2006; Westwood et al., 2006; Young et al., 2006; Allen et al., 2007; Bennett et al., 2007; Brennan et al., 2007; Drury et al., 2007; Eeles et al., 2007; Gulzar et al., 2007; Mak et al., 2007; Morad et al., 2007; Srinivasa et al., 2007; Tozer and Lugton, 2007; Williams et al., 2007; Washington State Department of Health, 2008; Williamson and LeBlanc, 2008; Evans et al., 2009; Gu and Warren, 2009; Little et al., 2009; Metcalfe et al., 2009; Mulsow et al., 2009; Schofield et al., 2009; Smith et al., 2009; Burton et al., 2010; Shields et al., 2010; Watts et al., 2010; Aarden et al., 2011; Gu et al., 2011; Hoppe, 2011; Burton, 2012; Hamblion et al., 2012; Pohjola et al., 2012; Orlando et al., 2013, 2014; Pujol et al., 2013; Long and Goldblatt, 2014; Lucci et al., 2014; Plunkett et al., 2014; Vickery et al., 2014; Bell et al., 2015; Kirke et al., 2015
Through population screening programs	Prenatal screening: Massie et al., 2000; Berkenstadt et al., 2007; Washington State Department of Health, 2008; McCann et al., 2009; Metcalfe et al., 2009; Kaufmann et al., 2011; Currier et al., 2012; Long and Goldblatt, 2014; Nesbitt et al., 2014; Newborn screening: Massie et al., 2000; The Genetic Services Plan for Wisconsin, 2002; Comeau et al., 2004; Basran et al., 2005; Calzolari and Baroncini, 2005; Hanley, 2005; Henry et al., 2005; Byck et al., 2006; Puryear et al., 2006; Therrell et al., 2006; Windmill and Windmill, 2006; Southern et al., 2007; Washington State Department of Health, 2008; Little et al., 2009; Metcalfe et al., 2009; Streetly et al., 2009; Thuret et al., 2010; Hoppe, 2011; Kaufmann et al., 2011; Mogayzel et al., 2014
**GENETIC COUNSELING**	
Medical geneticists	Brain et al., 2000, 2002; Gray et al., 2000; Harper et al., 2000; Pichert and Stahel, 2000; Donnai and Elles, 2001; Wonderling et al., 2001; Charron et al., 2002; Hartenbach et al., 2002; Campbell et al., 2003; Fry et al., 2003; Hopwood et al., 2003; Rowland et al., 2003; Salbert, 2003; Henriksson et al., 2004; Holloway et al., 2004; Calzolari and Baroncini, 2005; Epplein et al., 2005; Byck et al., 2006; Foretova et al., 2006; Gronwald et al., 2006; Mackay and Taylor, 2006; Reis et al., 2006; Windmill and Windmill, 2006; Bennett et al., 2007; Mak et al., 2007; Srinivasa et al., 2007; Williams et al., 2007; Washington State Department of Health, 2008; Evans et al., 2009; Little et al., 2009; Schofield et al., 2009; Burton et al., 2010; Speechley and Nisker, 2010; Gu et al., 2011; Burton, 2012; Hamblion et al., 2012; Pujol et al., 2013; Ramsden et al., 2013; Kirk et al., 2014; Long and Goldblatt, 2014; Lucci et al., 2014; Orlando et al., 2014
Genetic counselors	Bickerstaff et al., 2001; Donnai and Elles, 2001; Ekstein and Katzenstein, 2001; Wonderling et al., 2001; Lee et al., 2002; Gason et al., 2003; Hopwood et al., 2003; Salbert, 2003; Henriksson et al., 2004; Epplein et al., 2005; Henry et al., 2005; Byck et al., 2006; Mackay and Taylor, 2006; Ricker et al., 2006; Bennett et al., 2007, 2010; Drury et al., 2007; Eeles et al., 2007; Morad et al., 2007; Srinivasa et al., 2007; Williams et al., 2007; Coffey et al., 2008; Washington State Department of Health, 2008; Williamson and LeBlanc, 2008; Evans et al., 2009; Gu and Warren, 2009; Little et al., 2009; McCann et al., 2009; Mulsow et al., 2009; Schofield et al., 2009; Smith et al., 2009; Burton et al., 2010; Gu et al., 2011; Blumenfeld et al., 2012; Burton, 2012; Currier et al., 2012; Eble et al., 2013; Ramsden et al., 2013; Kirk et al., 2014; Long and Goldblatt, 2014; Nesbitt et al., 2014; Orlando et al., 2014
Other medical specialists	Pichert and Stahel, 2000; Charron et al., 2002; Hartenbach et al., 2002; Lee et al., 2002; Campbell et al., 2003; Fry et al., 2003; Hopwood et al., 2003; Holloway et al., 2004; Byck et al., 2006; Washington State Department of Health, 2008; Gu and Warren, 2009; Mulsow et al., 2009; Pujol et al., 2013; Nesbitt et al., 2014; Bell et al., 2015; Kirke et al., 2015
Pre-test counseling provided	Bickerstaff et al., 2001; Ekstein and Katzenstein, 2001; Anton-Culver et al., 2003; Henriksson et al., 2004; Allen et al., 2007; Williams et al., 2007; Evans et al., 2009; Blumenfeld et al., 2012; Pujol et al., 2013
Post-test counseling provided	Lena-Russo et al., 2002; The Genetic Services Plan for Wisconsin, 2002; Barlow-Stewart et al., 2003; Menkiszak et al., 2003; Rowland et al., 2003; Shepherd et al., 2003; Hanley, 2005; Foretova et al., 2006; Ricker et al., 2006; Brennan et al., 2007; Southern et al., 2007; Williams et al., 2007; Williamson and LeBlanc, 2008; Streetly et al., 2009; McGuire and Burke, 2011; Evans et al., 2012; Turcu et al., 2013; Amato et al., 2014
Nurses	Brain et al., 2000; Gray et al., 2000; Pichert and Stahel, 2000; Shepherd et al., 2001, 2003, 2014; Campbell et al., 2003; Fry et al., 2003; Gozdzik et al., 2005; Westwood et al., 2006; Allen et al., 2007; Bennett et al., 2007; Brennan et al., 2007; Tozer and Lugton, 2007; Washington State Department of Health, 2008; Speechley and Nisker, 2010; Burton, 2012; Kirk et al., 2014; Bell et al., 2015
Trained professionals	Anton-Culver et al., 2003; Barlow-Stewart et al., 2003; Allen et al., 2007; Gulzar et al., 2007; Tozer and Lugton, 2007; Washington State Department of Health, 2008; Kaufmann et al., 2011; Bell et al., 2015
Counseling offered in BRCA1/2 testing programs	Brain et al., 2000, 2002; Gray et al., 2000; Pichert and Stahel, 2000; Donnai and Elles, 2001; Wonderling et al., 2001; Hartenbach et al., 2002; Lee et al., 2002; Campbell et al., 2003; Fry et al., 2003; Hopwood et al., 2003; Menkiszak et al., 2003; Rowland et al., 2003; Henriksson et al., 2004; Holloway et al., 2004; Calzolari and Baroncini, 2005; Epplein et al., 2005; Foretova et al., 2006; Gronwald et al., 2006; Mackay and Taylor, 2006; Reis et al., 2006; Ricker et al., 2006; Westwood et al., 2006; Bennett et al., 2007; Brennan et al., 2007; Eeles et al., 2007; Gulzar et al., 2007; Srinivasa et al., 2007; Tozer and Lugton, 2007; Washington State Department of Health, 2008; Williamson and LeBlanc, 2008; Evans et al., 2009, 2012; Little et al., 2009; Mulsow et al., 2009; Smith et al., 2009; Speechley and Nisker, 2010; Eble et al., 2013; Pujol et al., 2013; Koeneman et al., 2014; Orlando et al., 2014; Bell et al., 2015; Slade et al., 2015
Counseling offered in Lynch syndrome testing programs	Pichert and Stahel, 2000; Wonderling et al., 2001; Hopwood et al., 2003; Henriksson et al., 2004; Epplein et al., 2005; Bennett et al., 2007; Mak et al., 2007; Williams et al., 2007; Schofield et al., 2009; Pujol et al., 2013; Orlando et al., 2014
**FAMILY HISTORY AND RISK ASSESSMENT**	
Medical geneticists	Brain et al., 2000, 2002; Gray et al., 2000; Pichert and Stahel, 2000; Donnai and Elles, 2001; Wonderling et al., 2001; Charron et al., 2002; Hartenbach et al., 2002; The Genetic Services Plan for Wisconsin, 2002; Hopwood et al., 2003; Salbert, 2003; Calzolari and Baroncini, 2005; Epplein et al., 2005; Foretova et al., 2006; Gronwald et al., 2006; Mackay and Taylor, 2006; Puryear et al., 2006; Reis et al., 2006; Windmill and Windmill, 2006; Mak et al., 2007; Srinivasa et al., 2007; Washington State Department of Health, 2008; Burton et al., 2010; Gu et al., 2011; Ramsden et al., 2013; Lucci et al., 2014
Other medical specialists	Brain et al., 2000, 2002; Gray et al., 2000; Pichert and Stahel, 2000; Bickerstaff et al., 2001; Ekstein and Katzenstein, 2001; Charron et al., 2002; Hartenbach et al., 2002; Anton-Culver et al., 2003; Hopwood et al., 2003; Epplein et al., 2005; Ricker et al., 2006; Brennan et al., 2007; Mak et al., 2007; Williams et al., 2007; Washington State Department of Health, 2008; Gu and Warren, 2009; Schofield et al., 2009; Smith et al., 2009; Blumenfeld et al., 2012; Burton, 2012; Orlando et al., 2013, 2014; Ramsden et al., 2013; Turcu et al., 2013; Amato et al., 2014; Long and Goldblatt, 2014; Plunkett et al., 2014; Shepherd et al., 2014; Vickery et al., 2014; Kirke et al., 2015
General practitioners	Pichert and Stahel, 2000; Wonderling et al., 2001; Hopwood et al., 2003; Epplein et al., 2005; Ricker et al., 2006; Brennan et al., 2007; Drury et al., 2007; Eeles et al., 2007; Mak et al., 2007; Srinivasa et al., 2007; Washington State Department of Health, 2008; Gu and Warren, 2009; Schofield et al., 2009; Burton, 2012; Orlando et al., 2013, 2014; Long and Goldblatt, 2014; Plunkett et al., 2014; Vickery et al., 2014; Bell et al., 2015; Kirke et al., 2015
Trained professionals	Brain et al., 2000, 2002; Gray et al., 2000; Wonderling et al., 2001; Lee et al., 2002; Campbell et al., 2003; Fry et al., 2003; Hopwood et al., 2003; Shepherd et al., 2003, 2014; Holloway et al., 2004; Epplein et al., 2005; Byck et al., 2006; Westwood et al., 2006; Young et al., 2006; Bennett et al., 2007; Berkenstadt et al., 2007; Brennan et al., 2007; Drury et al., 2007; Eeles et al., 2007; Gulzar et al., 2007; Srinivasa et al., 2007; Washington State Department of Health, 2008; Williamson and LeBlanc, 2008; Moeschler et al., 2009; Mulsow et al., 2009; Thuret et al., 2010; Orlando et al., 2013, 2014; Kirk et al., 2014; Long and Goldblatt, 2014; Vickery et al., 2014; Bell et al., 2015; Kirke et al., 2015
BRCA1/2 programs providing family history collection and risk assessment	Brain et al., 2000, 2002; Gray et al., 2000; Pichert and Stahel, 2000; Donnai and Elles, 2001; Wonderling et al., 2001; Hartenbach et al., 2002; Lee et al., 2002; Anton-Culver et al., 2003; Campbell et al., 2003; Fry et al., 2003; Hopwood et al., 2003; Menkiszak et al., 2003; Henriksson et al., 2004; Holloway et al., 2004; Calzolari and Baroncini, 2005; Epplein et al., 2005; Foretova et al., 2006; Gronwald et al., 2006; Mackay and Taylor, 2006; Ricker et al., 2006; Westwood et al., 2006; Young et al., 2006; Allen et al., 2007; Bennett et al., 2007; Brennan et al., 2007; Drury et al., 2007; Eeles et al., 2007; Gulzar et al., 2007; Srinivasa et al., 2007; Tozer and Lugton, 2007; Washington State Department of Health, 2008; Williamson and LeBlanc, 2008; Evans et al., 2009, 2012; Mulsow et al., 2009; Smith et al., 2009; Orlando et al., 2013, 2014; Koeneman et al., 2014
Lynch syndrome programs providing family history collection and risk assessment	Pichert and Stahel, 2000; Wonderling et al., 2001; Hopwood et al., 2003; Henriksson et al., 2004; Epplein et al., 2005; Bennett et al., 2007; Mak et al., 2007; Williams et al., 2007; Schofield et al., 2009, 2014; Orlando et al., 2013; Koeneman et al., 2014; Plunkett et al., 2014
**GENETIC TESTING AND INFORMED CONSENT**	
Medical geneticists	Brain et al., 2000, 2002; Gray et al., 2000; Harper et al., 2000; Pichert and Stahel, 2000; Donnai and Elles, 2001; Shepherd et al., 2001; Charron et al., 2002; The Genetic Services Plan for Wisconsin, 2002; Campbell et al., 2003; Fry et al., 2003; Hopwood et al., 2003; Rowland et al., 2003; Salbert, 2003; Henriksson et al., 2004; Holloway et al., 2004; Calzolari and Baroncini, 2005; Epplein et al., 2005; Byck et al., 2006; Foretova et al., 2006; Gronwald et al., 2006; Mackay and Taylor, 2006; Puryear et al., 2006; Reis et al., 2006; Windmill and Windmill, 2006; Bennett et al., 2007; Mak et al., 2007; Srinivasa et al., 2007; Williams et al., 2007; Washington State Department of Health, 2008; Evans et al., 2009; Metcalfe et al., 2009; Schofield et al., 2009; Burton et al., 2010; Speechley and Nisker, 2010; Aarden et al., 2011; Gu et al., 2011; Burton, 2012; Hamblion et al., 2012; Pujol et al., 2013; Ramsden et al., 2013; Amato et al., 2014; Kirk et al., 2014; Long and Goldblatt, 2014; Lucci et al., 2014; Orlando et al., 2014; Slade et al., 2015
Other medical specialists	Harper et al., 2000; Pichert and Stahel, 2000; Heath et al., 2001; Charron et al., 2002; Hartenbach et al., 2002; Lee et al., 2002; Lena-Russo et al., 2002; Hopwood et al., 2003; Menkiszak et al., 2003; Comeau et al., 2004; Byck et al., 2006; Berkenstadt et al., 2007; Southern et al., 2007; Eisinger, 2008; Washington State Department of Health, 2008; Gu and Warren, 2009; Little et al., 2009; Metcalfe et al., 2009; Mulsow et al., 2009; Streetly et al., 2009; Shields et al., 2010; Speechley and Nisker, 2010; Thuret et al., 2010; Aarden et al., 2011; Hoppe, 2011; Kaufmann et al., 2011; Pohjola et al., 2012; Pujol et al., 2013; Turcu et al., 2013; O'Brien et al., 2014; Bell et al., 2015; Kirke et al., 2015; Slade et al., 2015
Counselors not qualified in genetics	Harper et al., 2000; Bickerstaff et al., 2001; Donnai and Elles, 2001; Ekstein and Katzenstein, 2001; Heath et al., 2001; Hartenbach et al., 2002; Lee et al., 2002; The Genetic Services Plan for Wisconsin, 2002; Barlow-Stewart et al., 2003; Rowland et al., 2003; Salbert, 2003; Shepherd et al., 2003, 2014; Henriksson et al., 2004; Epplein et al., 2005; Gason et al., 2005; Byck et al., 2006; Mackay and Taylor, 2006; Ricker et al., 2006; Westwood et al., 2006; Allen et al., 2007; Bennett et al., 2007; Berkenstadt et al., 2007; Brennan et al., 2007; Drury et al., 2007; Eeles et al., 2007; Gulzar et al., 2007; Morad et al., 2007; Srinivasa et al., 2007; Tozer and Lugton, 2007; Coffey et al., 2008; Kaye, 2008; Washington State Department of Health, 2008; Williamson and LeBlanc, 2008; Evans et al., 2009, 2012; Gu and Warren, 2009; McCann et al., 2009; Metcalfe et al., 2009; Mulsow et al., 2009; Schofield et al., 2009; Smith et al., 2009; Burton et al., 2010; Speechley and Nisker, 2010; Aarden et al., 2011; Gu et al., 2011; McGuire and Burke, 2011; Blumenfeld et al., 2012; Burton, 2012; Currier et al., 2012; Eble et al., 2013; Ramsden et al., 2013; Bell et al., 2014; Kirk et al., 2014; Long and Goldblatt, 2014; Nesbitt et al., 2014; Orlando et al., 2014; Slade et al., 2015
Consent form required	Pichert and Stahel, 2000; Bickerstaff et al., 2001; Ekstein and Katzenstein, 2001; Shepherd et al., 2001; Charron et al., 2002; Lee et al., 2002; Lena-Russo et al., 2002; Barlow-Stewart et al., 2003; Gason et al., 2003; Menkiszak et al., 2003; Salbert, 2003; Henriksson et al., 2004; Kornreich et al., 2004; Calzolari and Baroncini, 2005; Foretova et al., 2006; Gronwald et al., 2006; Mackay and Taylor, 2006; Therrell et al., 2006; Bennett et al., 2007; Morad et al., 2007; Southern et al., 2007; Coffey et al., 2008; Eisinger, 2008; Washington State Department of Health, 2008; Evans et al., 2009, 2012; Gu and Warren, 2009; Schofield et al., 2009, 2014; Gu et al., 2011; Kaufmann et al., 2011; Pohjola et al., 2012; Eble et al., 2013; Ramsden et al., 2013; Amato et al., 2014; Long and Goldblatt, 2014; Vickery et al., 2014; Bell et al., 2015; Kirke et al., 2015
Genetic services integrated into healthcare systems requiring consent forms	Bickerstaff et al., 2001; Ekstein and Katzenstein, 2001; Shepherd et al., 2001; Lee et al., 2002; Lena-Russo et al., 2002; Menkiszak et al., 2003; Salbert, 2003; Henriksson et al., 2004; Kornreich et al., 2004; Calzolari and Baroncini, 2005; Foretova et al., 2006; Mackay and Taylor, 2006; Therrell et al., 2006; Morad et al., 2007; Southern et al., 2007; Coffey et al., 2008; Eisinger, 2008; Washington State Department of Health, 2008; Evans et al., 2009; Gu and Warren, 2009; Kaufmann et al., 2011; Eble et al., 2013; Amato et al., 2014; Long and Goldblatt, 2014; Schofield et al., 2014; Bell et al., 2015
Consent form prior to HBOC testing	Pichert and Stahel, 2000; Lee et al., 2002; Menkiszak et al., 2003; Henriksson et al., 2004; Calzolari and Baroncini, 2005; Foretova et al., 2006; Gronwald et al., 2006; Mackay and Taylor, 2006; Bennett et al., 2007; Eisinger, 2008; Evans et al., 2009, 2012; Eble et al., 2013
Consent form prior to Lynch syndrome testing	Pichert and Stahel, 2000; Henriksson et al., 2004; Bennett et al., 2007; Eisinger, 2008; Schofield et al., 2009, 2014
Consent form prior to CF testing	Bickerstaff et al., 2001; Ekstein and Katzenstein, 2001; Barlow-Stewart et al., 2003; Calzolari and Baroncini, 2005; Southern et al., 2007; Washington State Department of Health, 2008
**CASCADE TESTING**	
BRCA1/2	Brain et al., 2000, 2002; Gray et al., 2000; Pichert and Stahel, 2000; Donnai and Elles, 2001; Hartenbach et al., 2002; Lee et al., 2002; Anton-Culver et al., 2003; Hopwood et al., 2003; Rowland et al., 2003; Henriksson et al., 2004; Epplein et al., 2005; Foretova et al., 2006; Ricker et al., 2006; Bennett et al., 2007; Gulzar et al., 2007; Eisinger, 2008; Washington State Department of Health, 2008; Evans et al., 2009; Mulsow et al., 2009; Smith et al., 2009; Orlando et al., 2013; Pujol et al., 2013
Lynch syndrome	Pichert and Stahel, 2000; Hopwood et al., 2003; Henriksson et al., 2004; Epplein et al., 2005; Bennett et al., 2007; Mak et al., 2007; Eisinger, 2008; Schofield et al., 2009, 2014; Pujol et al., 2013
Familial hypercholesterolemia	Heath et al., 2001; Watts et al., 2010; Aarden et al., 2011; Burton, 2012; Bell et al., 2014, 2015; Kirk et al., 2014; Vickery et al., 2014
Newborn screening	Massie et al., 2000; Salbert, 2003; Comeau et al., 2004; Washington State Department of Health, 2008; Streetly et al., 2009

##### Access to genetic services

Direct access to genetic services was reported in 48 programs (Table 3), predominantly for BRCA1/2, Lynch syndrome (Pichert and Stahel, 2000; Hopwood et al., 2003; Henriksson et al., 2004; Epplein et al., 2005; Pujol et al., 2013), and newborn screening (Byck et al., 2006; Puryear et al., 2006; Little et al., 2009; Metcalfe et al., 2009). Patients were also referred to genetic services by a range of medical specialists (i.e., medical geneticists, surgeons, oncologists, obstetrician-gynecologists, pediatricians, gastroenterologists), as well as GPs.

Non-medical HCPs involved in patient referrals included nurses (Byck et al., 2006; Bennett et al., 2007; Drury et al., 2007; Eeles et al., 2007; Orlando et al., 2013; Long and Goldblatt, 2014; Shepherd et al., 2014; Vickery et al., 2014), genetic coordinators of local health departments and genetic counselors in regional genetic centers (Bickerstaff et al., 2001; Byck et al., 2006; Coffey et al., 2008), and midwives (Byck et al., 2006; Hamblion et al., 2012). Referrals to genetic services were also made by different categories of HCP engaged in population screening programs, such as prenatal and newborn screening (Table 3); hereditary breast and ovarian cancer (HBOC) screening (Washington State Department of Health, 2008; Little et al., 2009; Smith et al., 2009; Evans et al., 2012); colorectal cancer screening (Little et al., 2009; Schofield et al., 2009, 2014); population-based screening of Ashkenazi Jews (i.e., Tay-Sachs, CF, Fanconi anemia type C, Canavan disease, Gaucher type I) (Bach et al., 2001; Ekstein and Katzenstein, 2001; Barlow-Stewart et al., 2003; Kornreich et al., 2004; Gason et al., 2005; Washington State Department of Health, 2008); screening of Mediterranean and North African populations (i.e., beta-thalassemia, sickle cell) (Lena-Russo et al., 2002; Amato et al., 2014); and familial hypercholesterolemia (FH) (Aarden et al., 2011).

Regarding DTC services, a physician referral was not required in most cases and patients were self-referred. One exception was a virtual clinic requiring referrals from GPs or other medical specialists (Washington State Department of Health, 2008).

##### Genetic counseling

Pre- and post-test counseling were mainly provided by medical geneticists (Table 3); genetic counselors (including those working for DTC companies); other medical specialists; and GPs (Hopwood et al., 2003; Washington State Department of Health, 2008; Gu and Warren, 2009; Pujol et al., 2013). In some studies, only either pre-test or post-test counseling were provided by various professionals. Nurses and other trained professionals were also involved in counseling sessions.

Programs where pre- and post-test counseling were performed mostly comprised those offering testing for BRCA1/2 (52/59 programs) and Lynch syndrome (19/23 programs) (Table 3). The post-test consultation time was specified in a few genetic programs (Ekstein and Katzenstein, 2001; Hopwood et al., 2003; Epplein et al., 2005; Gronwald et al., 2006) and ranged between 45 (Hopwood et al., 2003; Gronwald et al., 2006) and 120 min (Hopwood et al., 2003). A post-clinic letter containing the result of the genetic test was sent to referring HCPs in some programs, but mostly to patients that underwent genetic testing for HBOC (Brain et al., 2002; Campbell et al., 2003; Fry et al., 2003; Hopwood et al., 2003; Holloway et al., 2004; Epplein et al., 2005; Reis et al., 2006; Ricker et al., 2006; Westwood et al., 2006; Young et al., 2006; Eeles et al., 2007; Gulzar et al., 2007; Tozer and Lugton, 2007); Lynch syndrome (Hopwood et al., 2003; Epplein et al., 2005; Williams et al., 2007; Schofield et al., 2009); FH (Heath et al., 2001; Bell et al., 2015); and newborn screening (Salbert, 2003; Byck et al., 2006). A face-to-face interview at the genetic service was not always offered to patients following receipt of the post-clinic letter.

Family history collection and risk assessment were provided prior to or during genetic counseling or medical examinations by medical geneticists (Table 3), other medical specialists and GPs. Non-medical HCPs such as genetic counselors, genetic associates and other trained professionals were also involved. The trained professionals were nurses engaged in different medical specialties (i.e., cancer genetics, the genetics of diabetes, and the genetics of cardiac conditions); family history workers (Holloway et al., 2004); health educators (Brain et al., 2000); social workers (Kirk et al., 2014); and administrative staff of the screening services (Tozer and Lugton, 2007; McCann et al., 2009).

Risk assessment was performed largely through questionnaires, computer programs and face-to-face interviews. Other tools reported in a few studies were screening tests (i.e., 1st or 2nd trimester prenatal tests, screening test for FH), medical records, death certificates, cancer registry, telephone counseling and physician referral letters. Family history collection and risk assessment were mostly performed in programs providing BRCA1/2 (52/59 programs), Lynch syndrome (21/23 programs) (Table 3), and CF testing (10/17 programs) (Bickerstaff et al., 2001; Donnai and Elles, 2001; Ekstein and Katzenstein, 2001; Byck et al., 2006; Berkenstadt et al., 2007; Drury et al., 2007; Washington State Department of Health, 2008; Blumenfeld et al., 2012; Currier et al., 2012; Long and Goldblatt, 2014).

##### Genetic testing and informed consent

Among medical HCPs, genetic testing was initiated principally by medical geneticists (Table 3); other medical specialists (e.g., pediatricians, surgeons, clinicians engaged in screening programs, etc.); and GPs (Heath et al., 2001; Hopwood et al., 2003; Berkenstadt et al., 2007; Washington State Department of Health, 2008; Gu and Warren, 2009; Metcalfe et al., 2009; Shields et al., 2010; Aarden et al., 2011; Hoppe, 2011; Pohjola et al., 2012; Pujol et al., 2013). Genetic testing was also initiated by non-medical HCPs such as genetic counselors, genetic specialist nurses, trained genetic service staff, midwives and counselors not qualified in genetics.

A consent form prior to genetic testing was explicitly required and reported in 29% (43/148) of the genetic programs (Table 3). Of these, 27 programs were integrated into the healthcare system and five programs were provided by the private sector (Schofield et al., 2009, 2014; Eble et al., 2013; Long and Goldblatt, 2014). A consent form was usually required prior to testing for HBOC, Lynch syndrome; CF; hemoglobinopathies (Bickerstaff et al., 2001; Lena-Russo et al., 2002; Kaufmann et al., 2011; Bell et al., 2014; Long and Goldblatt, 2014), and newborn screening (Salbert, 2003; Calzolari and Baroncini, 2005; Therrell et al., 2006; Washington State Department of Health, 2008). The countries with the highest number of genetic programs requiring a consent form prior to testing were Australia (Ekstein and Katzenstein, 2001; Barlow-Stewart et al., 2003; Gason et al., 2003; Schofield et al., 2009, 2014; Long and Goldblatt, 2014; Vickery et al., 2014; Bell et al., 2015; Kirke et al., 2015) and the UK (Bickerstaff et al., 2001; Shepherd et al., 2001; Mackay and Taylor, 2006; Bennett et al., 2007; Southern et al., 2007; Evans et al., 2009, 2012; Ramsden et al., 2013), both with 10 such programs, followed by the USA with eight programs (Ekstein and Katzenstein, 2001; Lee et al., 2002; Salbert, 2003; Kornreich et al., 2004; Therrell et al., 2006; Coffey et al., 2008; Washington State Department of Health, 2008; Eble et al., 2013), and France (Charron et al., 2002; Lena-Russo et al., 2002; Southern et al., 2007; Eisinger, 2008) and Italy (Calzolari and Baroncini, 2005; Southern et al., 2007; Amato et al., 2014), both with four programs.

Cascade testing on relatives of index cases (probands) was performed in several genetic programs, mainly for BRCA1/2 Lynch syndrome; FH testing (Table 3) and newborn screening. The genetic services contacted relatives either directly (Anton-Culver et al., 2003; Hopwood et al., 2003; Comeau et al., 2004; Bennett et al., 2007; Evans et al., 2009; Moeschler et al., 2009; Schofield et al., 2009; Burton et al., 2010; Aarden et al., 2011; Hoppe, 2011; Kaufmann et al., 2011; Shepherd et al., 2014; Vickery et al., 2014; Bell et al., 2015), via a physician (Burton et al., 2010), or through index cases who were asked to inform their relatives and suggest testing to them (Shepherd et al., 2001; Brain et al., 2002; Lee et al., 2002; Barlow-Stewart et al., 2003; Hopwood et al., 2003; Rowland et al., 2003; Ricker et al., 2006; Coffey et al., 2008; Evans et al., 2009; Mulsow et al., 2009; Streetly et al., 2009; Burton et al., 2010; Ramsden et al., 2013; Bell et al., 2015). In the study by Schofield et al. (2009), the relatives were contacted only after the death of the index case.

##### Follow-up services

Follow-up services were provided in several programs and the period of surveillance ranged from 12 months (Bell et al., 2014) to long-term (Byck et al., 2006; Puryear et al., 2006; Washington State Department of Health, 2008; Burton et al., 2010; Watts et al., 2010) or lifetime (Brain et al., 2000, 2002; Gray et al., 2000; Pichert and Stahel, 2000; Wonderling et al., 2001; Anton-Culver et al., 2003; Rowland et al., 2003; Comeau et al., 2004; Henriksson et al., 2004; Gozdzik et al., 2005; Henry et al., 2005; Gronwald et al., 2006; Young et al., 2006; Washington State Department of Health, 2008; Mogayzel et al., 2014). Specific recommendations were given to patients during the follow-up period according to the underlying genetic disorder and the patient's level of risk.

#### Genetic Service Delivery Models

The above analysis of genetic services and programs (I) and patient care pathways (II) laid the groundwork for the identification and classification of genetic service delivery models. Delivery models for the provision of genetic testing can be classified into five categories according to which healthcare professional plays the most prominent role in patient pathways to care: (i) genetic services led by geneticists; (ii) the primary care model; (iii) the medical specialist model; (iv) genetic services integrated into population screening programs; and (v) the DTC model (Table 4). The classification was obtained by matching each model provided by Battista et al. (2012) with all possible patient pathways described by Gu and Warren (2009); Gu et al. (2011) (Gu and Warren, 2009; Gu et al., 2011) or identified in other literature records. A detailed description of the models is reported below.

**Table 4:** Genetic service delivery models according to the roles of the healthcare professionals involved in patients' pathways to care.

**Pathway**	**Model I: genetic services led by geneticists**	**Model II: primary care model**	**Model III: medical specialist model**	**Model IV: genetic services integrated into population screening programs**	**Model V: direct to consumer (DTC)**
A	Patient-(GP)-Medical specialist-Counselor-Lab	Patient-(GP)-Counselor-Lab	Patient-(GP)-Medical specialist-Lab	Patient-(GP)-Medical specialist-Counselor-Lab	Patient-Lab
B	Patient-Counselor-Lab	Patient-GP-Lab	Patient-Medical specialist-Counselor-Lab	Patient-(GP)-Medical specialist-Lab	Patient-(GP)-Medical specialist-Counselor-Lab (virtual clinic)
C				Patient-Counselor-Lab	

*GP, General Practitioner*.

##### Model I: Genetic services led by geneticists

In this model the professional team may include medical geneticists, genetic counselors, and other healthcare professionals (e.g., genetic nurses). The professional team is responsible for risk assessment, counseling and testing of individuals or families affected or at risk of genetic disorders. Depending on the case, the genetic team collaborates with other medical specialists (e.g., oncologists, cardiologists, nephrologists, etc.) who could also be part of the genetic service (e.g., multidisciplinary genetic clinics). Classical examples of this model are genetic services for rare diseases. The access of patients to this model of genetic service may occur through two different pathways:

- Patient-GP or medical specialist-Counselor-Lab
- Patient-Counselor-Lab

The first pathway (Ia) occurs when a patient seeks medical assistance from a GP or any specialist doctor who then makes a referral to the genetic service where a genetic counselor or a medical geneticist can perform a risk assessment. If a genetic test is relevant and available, they may suggest genetic testing to the patient; then samples are collected, and tests are performed in the laboratory. Based on the results of the test, genetic counselors or medical geneticists recommend surveillance and/or intervention. Clinical management of genetic conditions may involve various medical specialists, other than geneticists (e.g., oncologists, cardiologists, nephrologists, endocrinologists, etc.). The second pathway (Ib) occurs when a patient, without a medical referral, contacts a genetic service where a genetic counselor or a medical geneticist can perform a risk assessment. Pathway Ib corresponds to pathway Ia from this point onward.

**Figure 3:**
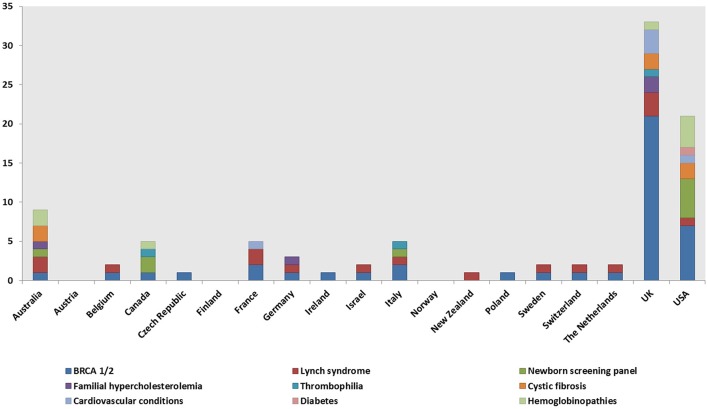
Model I: genetic services led by geneticists. Geographical distribution of the genetic tests.

Model I was identified in 74 genetic programs (Table 5) and pathway Ia was the most frequent. The model is common in the UK, the USA and Australia. The main genetic tests offered under Model I are BRCA1/2 (43 programs), Lynch syndrome (16 programs) and newborn screening panel (9 programs) (Figure 3).

**Table 5:** Genetic service delivery models and service evaluation.

**Models**	**References**
Model I: genetic services led by geneticists	Brain et al., 2000, 2002; Gray et al., 2000; Harper et al., 2000; Pichert and Stahel, 2000; Bickerstaff et al., 2001; Donnai and Elles, 2001; Heath et al., 2001; Wonderling et al., 2001; Charron et al., 2002; Hartenbach et al., 2002; The Genetic Services Plan for Wisconsin, 2002; Campbell et al., 2003; Fry et al., 2003; Hopwood et al., 2003; Rowland et al., 2003; Salbert, 2003; Henriksson et al., 2004; Holloway et al., 2004; Basran et al., 2005; Calzolari and Baroncini, 2005; Epplein et al., 2005; Henry et al., 2005; Byck et al., 2006; Foretova et al., 2006; Gronwald et al., 2006; Mackay and Taylor, 2006; Reis et al., 2006; Ricker et al., 2006; Young et al., 2006; Brennan et al., 2007; Eeles et al., 2007; Gulzar et al., 2007; Mak et al., 2007; Coffey et al., 2008; Washington State Department of Health, 2008; Williamson and LeBlanc, 2008; Evans et al., 2009; Gu and Warren, 2009; Little et al., 2009; Metcalfe et al., 2009; Moeschler et al., 2009; Mulsow et al., 2009; Schofield et al., 2009; Smith et al., 2009; Burton et al., 2010; Aarden et al., 2011; Gu et al., 2011; Burton, 2012; Hamblion et al., 2012; Eble et al., 2013; Pujol et al., 2013; Ramsden et al., 2013; Bell et al., 2014; Kirk et al., 2014; Long and Goldblatt, 2014; Lucci et al., 2014; Plunkett et al., 2014; Slade et al., 2015
UK	Brain et al., 2000, 2002; Gray et al., 2000; Harper et al., 2000; Bickerstaff et al., 2001; Donnai and Elles, 2001; Heath et al., 2001; Wonderling et al., 2001; Campbell et al., 2003; Fry et al., 2003; Hopwood et al., 2003; Holloway et al., 2004; Mackay and Taylor, 2006; Reis et al., 2006; Young et al., 2006; Brennan et al., 2007; Eeles et al., 2007; Gulzar et al., 2007; Mak et al., 2007; Evans et al., 2009; Burton et al., 2010; Burton, 2012; Hamblion et al., 2012; Ramsden et al., 2013; Kirk et al., 2014; Slade et al., 2015
USA	Hartenbach et al., 2002; The Genetic Services Plan for Wisconsin, 2002; Salbert, 2003; Epplein et al., 2005; Henry et al., 2005; Byck et al., 2006; Ricker et al., 2006; Coffey et al., 2008; Washington State Department of Health, 2008; Williamson and LeBlanc, 2008; Moeschler et al., 2009; Smith et al., 2009; Eble et al., 2013
Australia	Rowland et al., 2003; Metcalfe et al., 2009; Schofield et al., 2009; Bell et al., 2014; Long and Goldblatt, 2014
Model II: primary care model	Heath et al., 2001; Shepherd et al., 2001; Reis et al., 2006; Westwood et al., 2006; Allen et al., 2007; Bennett et al., 2007, 2010; Drury et al., 2007; Eeles et al., 2007; Srinivasa et al., 2007; Tozer and Lugton, 2007; Washington State Department of Health, 2008; Gu and Warren, 2009; Metcalfe et al., 2009; Shields et al., 2010; Aarden et al., 2011; Gu et al., 2011; Hoppe, 2011; Hamblion et al., 2012; Orlando et al., 2013, 2014; Pujol et al., 2013; Vickery et al., 2014; Kirke et al., 2015; Slade et al., 2015
UK	Heath et al., 2001; Shepherd et al., 2001; Reis et al., 2006; Westwood et al., 2006; Allen et al., 2007; Bennett et al., 2007, 2010; Drury et al., 2007; Eeles et al., 2007; Srinivasa et al., 2007; Tozer and Lugton, 2007; Shields et al., 2010; Hamblion et al., 2012; Slade et al., 2015
USA	Washington State Department of Health, 2008; Hoppe, 2011; Orlando et al., 2013, 2014
Model III: medical specialist model	Brain et al., 2000, 2002; Gray et al., 2000; Harper et al., 2000; Heath et al., 2001; Charron et al., 2002; Lee et al., 2002; Anton-Culver et al., 2003; Hopwood et al., 2003; Menkiszak et al., 2003; Shepherd et al., 2003, 2014; Gozdzik et al., 2005; Byck et al., 2006; Bennett et al., 2007, 2010; Eeles et al., 2007; Morad et al., 2007; Williams et al., 2007; Washington State Department of Health, 2008; Gu and Warren, 2009; Mulsow et al., 2009; Shields et al., 2010; Speechley and Nisker, 2010; Thuret et al., 2010; Watts et al., 2010; Aarden et al., 2011; Gu et al., 2011; Burton, 2012; Pohjola et al., 2012; Pujol et al., 2013; Ramsden et al., 2013; Turcu et al., 2013; Amato et al., 2014; Koeneman et al., 2014; Lucci et al., 2014; O'Brien et al., 2014; Schofield et al., 2014; Vickery et al., 2014; Kirke et al., 2015
UK	Brain et al., 2000, 2002; Gray et al., 2000; Harper et al., 2000; Heath et al., 2001; Shepherd et al., 2003, 2014; Bennett et al., 2007, 2010; Eeles et al., 2007; Williams et al., 2007; Shields et al., 2010; Aarden et al., 2011; Burton, 2012; Ramsden et al., 2013; Turcu et al., 2013
USA	Lee et al., 2002; Anton-Culver et al., 2003; Byck et al., 2006; Washington State Department of Health, 2008
Australia	Watts et al., 2010; Schofield et al., 2014; Vickery et al., 2014; Bell et al., 2015; Kirke et al., 2015
France	Charron et al., 2002; Hopwood et al., 2003; Thuret et al., 2010; Pujol et al., 2013
Model IV: genetic services integrated into population screening programs	Massie et al., 2000; Bach et al., 2001; Ekstein and Katzenstein, 2001; Lena-Russo et al., 2002; Barlow-Stewart et al., 2003; Gason et al., 2003, 2005; Comeau et al., 2004; Kornreich et al., 2004; Basran et al., 2005; Hanley, 2005; Henry et al., 2005; Byck et al., 2006; Puryear et al., 2006; Therrell et al., 2006; Windmill and Windmill, 2006; Berkenstadt et al., 2007; Southern et al., 2007; Washington State Department of Health, 2008; Little et al., 2009; McCann et al., 2009; Metcalfe et al., 2009; Schofield et al., 2009, 2014; Smith et al., 2009; Streetly et al., 2009; Thuret et al., 2010; Aarden et al., 2011; Hoppe, 2011; Kaufmann et al., 2011; Blumenfeld et al., 2012; Currier et al., 2012; Evans et al., 2012; Hamblion et al., 2012; Amato et al., 2014; Long and Goldblatt, 2014; Mogayzel et al., 2014; Nesbitt et al., 2014
USA	Bach et al., 2001; Ekstein and Katzenstein, 2001; The Genetic Services Plan for Wisconsin, 2002; Comeau et al., 2004; Kornreich et al., 2004; Henry et al., 2005; Byck et al., 2006; Puryear et al., 2006; Therrell et al., 2006; Windmill and Windmill, 2006; Washington State Department of Health, 2008; Smith et al., 2009; Hoppe, 2011; Blumenfeld et al., 2012; Currier et al., 2012; Mogayzel et al., 2014
Australia	Massie et al., 2000; Ekstein and Katzenstein, 2001; Barlow-Stewart et al., 2003; Gason et al., 2003, 2005; Metcalfe et al., 2009; Schofield et al., 2009, 2014; Long and Goldblatt, 2014
UK	Southern et al., 2007; McCann et al., 2009; Streetly et al., 2009; Evans et al., 2012; Hamblion et al., 2012; Nesbitt et al., 2014
Genetic service evaluation	Gray et al., 2000; Harper et al., 2000; Pichert and Stahel, 2000; Bickerstaff et al., 2001; Donnai and Elles, 2001; Wonderling et al., 2001; Brain et al., 2002; Charron et al., 2002; Lee et al., 2002; Lena-Russo et al., 2002; Barlow-Stewart et al., 2003; Campbell et al., 2003; Fry et al., 2003; Gason et al., 2003; Comeau et al., 2004; Henriksson et al., 2004; Holloway et al., 2004; Kornreich et al., 2004; Basran et al., 2005; Gronwald et al., 2006; Ricker et al., 2006; Berkenstadt et al., 2007; Drury et al., 2007; Eeles et al., 2007; Gulzar et al., 2007; Mak et al., 2007; Morad et al., 2007; Southern et al., 2007; Srinivasa et al., 2007; Tozer and Lugton, 2007; Williams et al., 2007; Coffey et al., 2008; McCann et al., 2009; Thuret et al., 2010; Watts et al., 2010; Kaufmann et al., 2011; Evans et al., 2012; Pujol et al., 2013; Ramsden et al., 2013; Bell et al., 2014, 2015; Orlando et al., 2014; Plunkett et al., 2014; Schofield et al., 2014; Kirke et al., 2015; Slade et al., 2015

##### Model II: Primary care model

In this model, a prominent role is played by primary care units in which GPs have specific genetic skills and can undertake an initial risk assessment using standardized referral guidelines. In some cases, GPs refer patients who are categorized as “high risk” to genetic services, while in other cases they can deliver genetic counseling, request genetic testing, and interpret the results. Therefore, in this model, there are two possible patient pathways:

- Patient-GP-Counselor-Lab
- Patient-GP-Lab

Pathway IIa occurs when a patient contacts a GP who undertakes the initial risk assessment and then makes referrals to a genetic service, where a genetic counselor or a medical geneticist can perform counseling and suggest genetic testing to the patient. A variation of pathway IIa was found in the GSPP Report 2008 (Washington State Department of Health, 2008), in which only post-counseling was offered to patients. Thus, patients were seen by the genetic counselor only after the genetic test: Patient-GP-Lab-Counselor. Pathway IIb occurs when a patient contacts a GP who can perform the risk assessment, undertake counseling and suggest genetic testing.

**Figure 4:**
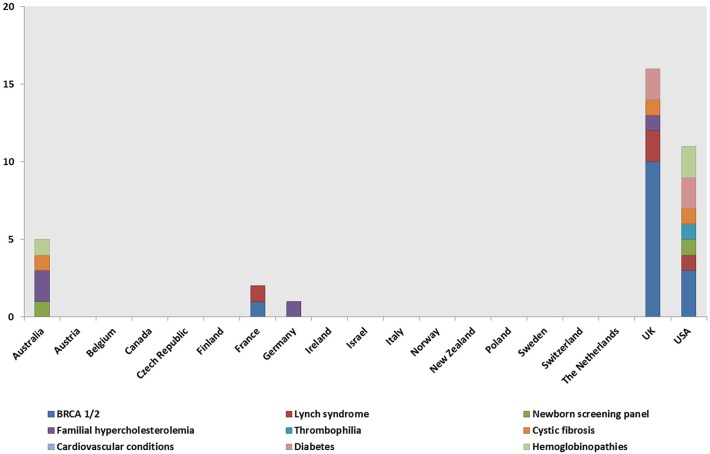
Model II: primary care model. Geographical distribution of the genetic tests.

Model II, most frequently pathway IIa, was identified in 30 genetic programs (Table 5). The model is prevalent in the UK and in the USA. The main genetic tests offered under Model II are BRCA1/2 (14 programs), Lynch syndrome, FH, and diabetes (four programs each) (Figure 4).

##### Model III: Medical specialist model

In this model, genetic tests can be requested directly by medical specialists (e.g., oncologists, cardiologists, neurologists, etc.) who may be able to manage patients with genetic disorders without consulting medical geneticists. Thus, a medical specialist may request genetic testing, communicate genetic test results to patients and families and set up treatment with or without consulting a medical geneticist. There are two main patient pathways in Model III:

- Patient-(GP)-Medical specialist-Lab
- Patient-(GP)-Medical specialist-Counselor-Lab

Pathway IIIa occurs when a patient contacts (with or without a GP referral) a medical specialist who performs a risk assessment, undertakes genetic counseling, and suggests genetic testing. Two variations of pathway IIIa have been identified in the studies of Shepherd et al. (2014) and Schofield et al. (2014). In Shepherd et al. (2014), patients were referred for maturity onset diabetes of the young (MODY; also known as monogenic diabetes) genetic testing by a genetic diabetic nurse (GDN) working in a diabetes clinical team. The GDN also guided the management and treatment of patients with monogenic diabetes and provided ongoing support to families and clinicians. The related pathway is: Patient-(GP)-Medical specialist/GDN-Lab. In the study by Schofield et al. (2014), medical specialists (i.e., oncologists, surgeons) requested Lynch syndrome screening tests for all newly diagnosed colorectal cancer patients and referred all patients with positive results to genetic services for counseling and possible germline testing. The related pathway is: Patient-Medical specialist-Lab (screening) -Counselor-Lab (genetic testing). In pathway IIIb, a patient contacts (with or without a GP referral) a medical specialist who undertakes the initial risk assessment and then requests counseling, collaborating with the medical geneticist or genetic counselor in the management of the patient.

**Figure 5:**
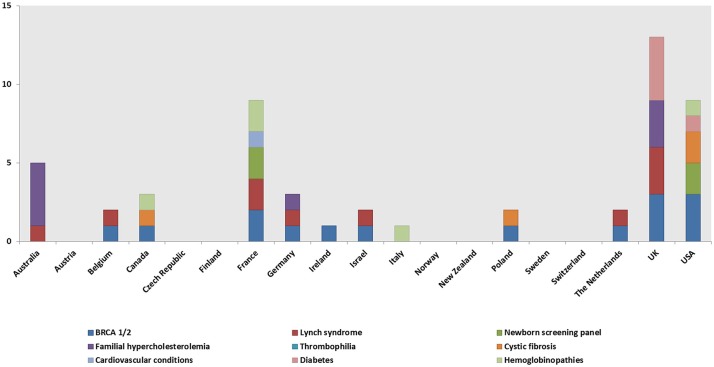
Model III: medical specialist model. Geographical distribution of the genetic tests.

Model III was identified in 54 genetic programs (Table 5). The associated pathways IIIa and IIIb were equally distributed in the programs. The model is common in the UK, the USA, Australia and France. The main genetic tests offered under Model III are BRCA1/2 (15 programs), Lynch syndrome (10 programs) and FH (eight programs) (Figure 5).

##### Model IV: Genetic services integrated into population screening programs

In this model, genetic services are provided within organized population screening programs (e.g., newborn screening, cervical cancer screening, HBOC screening, colorectal cancer screening, Ashkenazi Jewish genetic screening, etc.). There are three possible patient pathways in Model IV:

- Patient-GP/Medical specialist-Counselor-Lab
- Patient-GP/Medical specialist-Lab
- Patient-Counselor-Lab

Pathway IVa occurs when a patient takes part in a population-based screening program; a physician (or another HCP) involved in the screening program can perform an initial risk assessment and refer the patient for genetic counseling. The genetic counselor or medical geneticist can undertake counseling, suggest genetic testing and, based on the results of the test, can recommend surveillance and/or intervention. A variation of the IVa pathway was found in one record (Washington State Department of Health, 2008), in which only post-test counseling was offered to patients (Patient-GP/Medical specialist-Lab-Counselor). In pathway IVb, a patient takes part in a population-based screening program; a physician (or another HCP) involved in the screening program can perform risk assessment, undertake counseling, and suggest genetic testing. Based on the results of the test, the physician can recommend surveillance and/or intervention. In pathway IVc, a patient contacts a genetic counselor or a medical geneticist who can undertake counseling, suggest genetic testing and, based on the results of the test, can suggest surveillance through available population-based screening programs and/or intervention.

**Figure 6:**
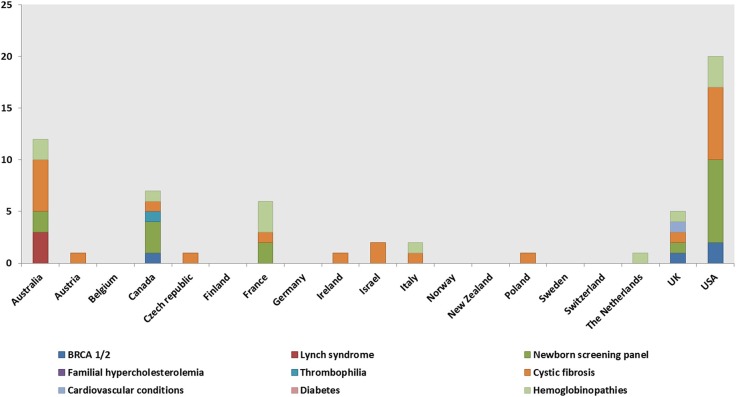
Model IV: genetic services integrated into population screening programs. Geographical distribution of the genetic tests.

Model IV was identified in 44 genetic programs (Table 5). The most frequent patient pathways were IVa and IVb. Model IV is common in the USA, Australia and in the UK. The main genetic tests offered under Model IV are CF (22 programs), newborn screening panel (16 programs), and hemoglobinopathies screening (12 programs) (Figure 6).

##### Model V: Direct to consumer (DTC)

In this model, private companies offer genetic services, typically through websites. The pathways associated with Model V are:

- Patient-Lab-Counselor
- Patient-GP/Medical specialist-Counselor-Lab (virtual clinic)

Healthcare professionals are usually not involved in the process and medical referrals are not required for genetic testing through DTC companies; thus, patients are self-referred. Furthermore, the companies usually do not offer risk assessment and genetic counseling. In pathway Va, patients purchase the test, take their own sample at home, send it to the lab, and receive the results directly. In contrast, a web-based virtual genetics clinic (i.e., DNA DIRECT) operating pathway Vb requires referrals from GPs or other medical specialists, offers risk assessment, pre- and post-test genetic counseling performed by genetic counselors, and genetic testing that can be requested by genetic counselors or medical specialists (Washington State Department of Health, 2008). Some DTC companies only offer post-test genetic counseling (Kaye, 2008; McGuire and Burke, 2011). Model V was identified in five genetic programs available in the UK (Kaye, 2008), the USA (Washington State Department of Health, 2008; McGuire and Burke, 2011), and in New Zealand (Gu and Warren, 2009; Gu et al., 2011). The genetic tests offered under Model V were not well defined.

#### Genetic Service Evaluation

Evidence of efficacy and effectiveness (i.e., guidelines and recommendations of scientific societies, health economic evaluations, feasibility studies) were reported for numerous genetic programs (Table 5). The cost-effectiveness of the interventions was reported for nine genetic programs (Gulzar et al., 2007; Morad et al., 2007; Thuret et al., 2010; Kaufmann et al., 2011; Bell et al., 2014, 2015; Vickery et al., 2014; Kirke et al., 2015) and a feasibility analysis, intended as an evaluation of a proposed project to determine if it is technically and economically feasible, was reported for 11 programs (Holloway et al., 2004; Gronwald et al., 2006; Reis et al., 2006; Westwood et al., 2006; Moeschler et al., 2009; Streetly et al., 2009; Kaufmann et al., 2011; Evans et al., 2012; Pohjola et al., 2012; Bell et al., 2015).

The genetic conditions and the related tests identified in the review are presented as a three-tier classification (Tables 6–8) according to the Centers for Disease Control and Prevention's (CDC's) Office of Public Health Genomics evidence-based classification of genomic applications. Tier 1 encompasses genomic applications supported by evidence for implementation in practice; Tier 2 includes genetic applications with insufficient evidence supporting their routine implementation in practice but which may be useful for informed decision making; and Tier 3 comprises genetic applications lacking evidence or with irrelevant synthesized evidence, which are therefore not ready for routine implementation in practice, or have synthesized evidence that supports recommendations against or discourages use (Centers for Disease Control Prevention, 2018).

**Table 6:** Genetic tests identified in the literature studies and classified in Tier 1 according to the Center of Disease Control and Prevention (CDC).

**Disease/disorder**	**Test or application**	**Intended use**	**N programs**	**References**
BRCA-related cancer; hereditary breast and ovarian cancer	Family history of known breast/ovarian cancer (1st or 2nd degree relative); personal history of any tumor type where profiling showed BRCA1/2 pathogenic mutation	Risk prediction for referral to further risk assessment, genetic counseling and possibly genetic testing	59	Brain et al., 2000, 2002; Gray et al., 2000; Pichert and Stahel, 2000; Donnai and Elles, 2001; Wonderling et al., 2001; Hartenbach et al., 2002; Lee et al., 2002; Anton-Culver et al., 2003; Campbell et al., 2003; Fry et al., 2003; Hopwood et al., 2003; Menkiszak et al., 2003; Rowland et al., 2003; Henriksson et al., 2004; Holloway et al., 2004; Calzolari and Baroncini, 2005; Epplein et al., 2005; Foretova et al., 2006; Gronwald et al., 2006; Mackay and Taylor, 2006; Reis et al., 2006; Ricker et al., 2006; Westwood et al., 2006; Young et al., 2006; Allen et al., 2007; Bennett et al., 2007; Brennan et al., 2007; Drury et al., 2007; Eeles et al., 2007; Gulzar et al., 2007; Srinivasa et al., 2007; Tozer and Lugton, 2007; Eisinger, 2008; Washington State Department of Health, 2008; Williamson and LeBlanc, 2008; Evans et al., 2009, 2012; Little et al., 2009; Mulsow et al., 2009; Smith et al., 2009; Speechley and Nisker, 2010; Eble et al., 2013; Orlando et al., 2013, 2014; Pujol et al., 2013; Koeneman et al., 2014; Slade et al., 2015
Lynch syndrome	Various strategies (i.e., family history of known cases of Lynch syndrome, newly diagnosed colorectal cancer)	Diagnostic, screening, and cascade testing of relatives	19	Pichert and Stahel, 2000; Wonderling et al., 2001; Hopwood et al., 2003; Epplein et al., 2005; Bennett et al., 2007; Gulzar et al., 2007; Mak et al., 2007; Eisinger, 2008; Pujol et al., 2013; Koeneman et al., 2014; Plunkett et al., 2014; Schofield et al., 2014
Familial hypercholesterolemia	DNA testing and LDL-C concentration measurement	Cascade testing of relatives of people diagnosed with FH	11	Heath et al., 2001; Burton et al., 2010; Watts et al., 2010; Aarden et al., 2011; Burton, 2012; Bell et al., 2014; Kirk et al., 2014; Vickery et al., 2014; Kirke et al., 2015
Symptoms and signs of disease suggesting specific causes of hypertrophic cardiomyopathy	Genetic testing	Confirm diagnosis of hypertrophic cardiomyopathy	1	Charron et al., 2002
Newborn and children screening (e.g., cystic fibrosis, hemoglobinopathies, critical congenital heart disease, hearing loss, etc.)	Newborn screening panel for 31 conditions; screening in minors for other conditions	Screening	36	Massie et al., 2000; Bickerstaff et al., 2001; Donnai and Elles, 2001; Ekstein and Katzenstein, 2001; Lena-Russo et al., 2002; The Genetic Services Plan for Wisconsin, 2002; Barlow-Stewart et al., 2003; Comeau et al., 2004; Kornreich et al., 2004; Basran et al., 2005; Calzolari and Baroncini, 2005; Gozdzik et al., 2005; Hanley, 2005; Henry et al., 2005; Byck et al., 2006; Puryear et al., 2006; Therrell et al., 2006; Windmill and Windmill, 2006; Drury et al., 2007; Southern et al., 2007; Washington State Department of Health, 2008; Little et al., 2009; Metcalfe et al., 2009; Streetly et al., 2009; Speechley and Nisker, 2010; Thuret et al., 2010; Hoppe, 2011; Kaufmann et al., 2011; Amato et al., 2014; Long and Goldblatt, 2014; Lucci et al., 2014; Mogayzel et al., 2014

According to the aforementioned criteria, most genetic programs identified in the review are included under Tier 1; specifically, these are genetic programs for HBOC, Lynch syndrome, FH, hypertrophic cardiomyopathy, and newborn screening (Table 6). Thirty-five genetic programs offering testing for various disorders, including Lynch syndrome under specific circumstances, are classified as Tier 2 (Table 7). Twenty-seven genetic programs offering not-yet-recommended genetic tests for various conditions (e.g., surfactant dysfunction, mitochondrial disease, cardiovascular conditions, type 2 diabetes) are reported as Tier 3 (Table 8). The tables with the three-tier classification (Tables 6–8) do not comprise all genetic programs identified in the review as the circumstances under which some tests were provided and the genetic conditions were not well specified (e.g., neuropathies, endocrine and metabolic disorders, etc.).

**Table 7:** Genetic tests identified in the literature studies and classified in Tier 2 according to the Center of Disease Control and Prevention (CDC).

**Disease/disorder**	**Test or application**	**Intended use**	**N programs**	**References**
Lynch syndrome	Testing for Lynch syndrome based only on family history (patients meeting revised Bethesda guidelines or Amsterdam criteria)	Diagnostic, screening	3	Henriksson et al., 2004; Eisinger, 2008; Orlando et al., 2014
Colorectal cancer in patient with 1st or 2nd degree relatives with Lynch syndrome related cancer at any age	Testing for Lynch syndrome	Diagnostic, screening	6	Anton-Culver et al., 2003; Epplein et al., 2005; Williams et al., 2007; Schofield et al., 2009; Orlando et al., 2013
Single gene disorders and chromosomal abnormalities	Various genetic tests without formal evidence synthesis and reviews by evidence panels	Diagnosis, management, carrier testing	22	Harper et al., 2000; Bach et al., 2001; Ekstein and Katzenstein, 2001; Barlow-Stewart et al., 2003; Gason et al., 2003, 2005; Salbert, 2003; Basran et al., 2005; Berkenstadt et al., 2007; Morad et al., 2007; Coffey et al., 2008; Washington State Department of Health, 2008; Williamson and LeBlanc, 2008; Little et al., 2009; Metcalfe et al., 2009; Speechley and Nisker, 2010; Hoppe, 2011; Blumenfeld et al., 2012; Burton, 2012; Currier et al., 2012; Ramsden et al., 2013; Amato et al., 2014
Lipid screening in infants, children, adolescents, or young adults (up to 20 years)	Family history relevant to dyslipidemia (otherwise undefined)	Risk prediction	1	Burton, 2012
Skin cancer screening in adults	Family history of skin cancer	Risk prediction	1	Henriksson et al., 2004
Prostate cancer	Tumor gene expression analysis	Risk prediction, management	2	Henriksson et al., 2004; Epplein et al., 2005

**Table 8:** Genetic tests identified in the literature studies and classified in Tier 3 according to the Center of Disease Control and Prevention (CDC).

**Disease/disorder**	**Test or application**	**Intended use**	**N programs**	**References**
Common diseases (e.g., cardiovascular conditions, type 2 diabetes, hereditary hemochromatosis)	Test for various genetic risk factors	Risk assessment	20	Donnai and Elles, 2001; Shepherd et al., 2001, 2003, 2014; Calzolari and Baroncini, 2005; Epplein et al., 2005; Brennan et al., 2007; Drury et al., 2007; Washington State Department of Health, 2008; Bennett et al., 2010; Shields et al., 2010; Burton, 2012; Orlando et al., 2013; Kirk et al., 2014; Kirke et al., 2015; National Library of Medicine, 2018
Various conditions (e.g., Fanconi anemia, surfactant dysfunction, mitochondrial diseases, intellectual disability, hereditary retinal diseases)	Panel of genes	Risk assessment, disease prevention	7	Ekstein and Katzenstein, 2001; Salbert, 2003; Moeschler et al., 2009; Hamblion et al., 2012; Pohjola et al., 2012; Turcu et al., 2013; Nesbitt et al., 2014; O'Brien et al., 2014

### Risk of Bias

We undertook measures to minimize the risk of bias. To avoid reporting bias, the review was conducted in accordance with a written protocol, which is published in *Frontiers in Public Health 2017*; volume 5, article 223 (Unim et al., 2017) and protocol deviations did not occur. Also, the review adheres to the PRISMA statement that defines the content of a review protocol (Liberati et al., 2009).

Different research strategies were employed to limit evidence selection bias and identify all relevant studies (i.e., non-systematic research, systematic review, multiple electronic resources, and manual searches of references). Concerning quality indicators, the studies are assessed in terms of reported effectiveness, cost-effectiveness, feasibility data, or evidence-based guidelines.

There are no competing interests and the funder had no role in the study design, data collection and analysis, or decision to publish.

## Discussion

### Summary of Main Findings

Although some genetic tests with insufficient evidence of clinical utility and validity are offered to the general population, most genetic tests identified in the review have considerable evidence of efficacy and cost-effectiveness and are ready for full implementation in clinical and public health practice. Leading examples of genetic tests with such specifications, and included in Tier 1, are BRCA1/2 genetic testing, genetic screening for Lynch syndrome, and FH. However, not all programs offering these three tests can be deemed equivalent or recommended considering the target population. Economic evaluations of genetic applications recognize three categories of BRCA1/2 genetic testing programs as cost-effective: (i) population-based screening among Ashkenazi Jews; (ii) family history-based screening, although methods on how to select high-risk women from the general population and the related cost are not detailed in literature studies; and (iii) cancer-based genetic screening, which includes tools for the identification of affected women at higher risk of inherited breast and ovarian cancers (D'Andrea et al., 2016). In the case of Lynch syndrome and FH, colorectal cancer-based universal screening programs or those targeting individuals <70 years old (Di Marco et al., 2018), and cascade screening of FH offered to relatives of index cases, are cost-effective (Rosso et al., 2017a). As a general approach, genomic applications should be evaluated rigorously prior to their introduction into clinical and public health practice by adapting the Health Technology Assessment framework for the evaluation of new technologies (Pitini et al., 2018). Those applications with proven efficacy and cost-effectiveness should be implemented in healthcare systems and made available to all citizens, as part of their right to safe and quality healthcare.

Despite the evidence supporting the use of specific genetic and genomic applications, there is a risk that they will not be implemented or will be implemented haphazardly (Burke et al., 2006). One of the primary factors limiting the successful implementation of genomic discoveries into routine clinical and public health practice is the lack of expertise in medical genetics (Henriksson et al., 2004; Byck et al., 2006; Ricker et al., 2006; Westwood et al., 2006; Bennett et al., 2007, 2010; Drury et al., 2007; Srinivasa et al., 2007; Washington State Department of Health, 2008; Gu and Warren, 2009; Pujol et al., 2013; Kirke et al., 2015). Lack of or limited knowledge, competency, and confidence of healthcare professionals in providing genetic risk assessments, genetic counseling, and referrals to clinical genetic centers can be overcome through proper information dissemination, education, and training activities. Another important barrier to implementation is related to funding for genomic research, which is public in most countries. The amount of funding provided, and the subsequent allocation of funds vary according to the healthcare budget and research priorities in each setting (Pohlhaus and Cook-Deegan, 2008). This leads to differences in the development and availability of genetic technologies across geographic regions. Collaborations between government health agencies, genetic service providers, and universities, nationally as well as internationally, in genomic research are necessary for the identification of priorities in research funding and the sustainability of genomic technologies.

Along with economic issues, national policies governing the use of genomic applications also affect the proper implementation of genetic discoveries in mainstream medicine. Italy is the only country, among those considered in the study, with a national plan for PHG; this recommends intervention strategies and concrete actions to the Italian Regions to develop and/or empower an understanding of predictive genomic applications, and to implement new technologies according to the principles of evidence-based medicine (Conferenza Stato Regioni, 2013). In addition, a national plan for innovation of the health system based on omics sciences, focused on the effectiveness and sustainability of genomic applications, was approved in Conferenza Stato Regioni (2017). In the USA, genetic services are regulated at both the federal (by the Food and Drug Administration according to the Clinical Laboratory Improvement Amendments) and state levels (Washington State Department of Health, 2008; McGuire and Burke, 2011) leading to substantial differences across the country. For instance, the use of genetic information in health insurance, embryonic and fetal research, and licensing of genetic counselors are not regulated in all states (Washington State Department of Health, 2008). The development of genetic applications should be accompanied by appropriate and uniform legislative oversight that can set quality standards, evaluate performance, and monitor outcomes of services nationwide.

DTC genetic testing legislation also varies across different settings (Kaye, 2008; Washington State Department of Health, 2008; Gu and Warren, 2009; Gu et al., 2011; McGuire and Burke, 2011). The challenge for policy makers is to develop a regulatory approach that will prevent potential risks resulting from unsupervised genetic testing (e.g., misinterpretation of genetic test results, distress, anxiety, major burden of healthcare practitioners and the healthcare system), while respecting individual freedom and the free market.

The present systematic review enabled the classification of genetic programs into five genetic service delivery models, according to which healthcare professionals play the most prominent roles in patient care pathways. Genetic services led by geneticists correspond to the “classic” model of genetic services (e.g., for rare diseases) provided mainly by geneticists; this is still the most common model of delivery. However, genetic applications are increasingly utilized by a wide range of healthcare professionals who are involved to various degrees in patient management (e.g., different medical specialists, nurses, technicians, midwives, social workers, and so on). More recently developed professional roles (i.e., genetic counselors, genetic associates, genetic nurses) have also been identified in several settings where they are vital in supporting clinicians in multidisciplinary teams. This is particularly evident in genetic services led by medical specialists, which is the second most common model of delivery. Genetic services are also progressively integrated into population-based screening programs. The review by Battista et al. (2012) reported on two early examples of this model, namely prenatal and newborn screening programs, while the present study identified more than 40 genetic testing programs integrated into population-based screening activities (i.e., CF and HBOC in Ashkenazi Jews, hemoglobinopathies in Mediterranean and North African populations). Although the integration of genetic testing services and screening programs is still at an early phase and not yet widely distributed, it underlines current efforts to strengthen the PHG framework, which represents an integrated system where genetic medicine is combined with health promotion and disease prevention activities. Efforts have also been made to integrate genetic knowledge into primary healthcare, but the primary care model is one of the least represented in the review. This could be because the primary care physicians providing the genetic services lack the relevant knowledge and skills. In fact, GPs represent the professional category that was least likely to have a genetic background compared to other healthcare professionals in the review. Battista et al. (2012) considered the primary care model “as the first step favoring the gradual introduction of integrated genetic services” and maintained high expectations for this model. The primary care model could be considered a pioneer of integrated services, but it has certainly been overshadowed by the medical specialist model in recent years. Regarding DTC services, only five programs were identified in the review, but the model should be much more common considering the easy access to genetic testing offered by commercial companies and the increasing tendency to purchase medical products through the internet.

BRCA1/2 and Lynch syndrome testing were the most frequently offered genetic tests. The most cost-effective BRCA1/2 tests, as well as family history-based screening and cancer-based genetic screening, are all delivered predominantly via genetic services led by geneticists, followed by the medical specialist and the primary care models. Furthermore, the BRCA1/2 population-based screening among Ashkenazi Jews is provided by physicians involved in population screening programs and referrals are carried out by different medical specialists. Lynch syndrome testing, including the cost-effective strategies, is mostly delivered by the geneticists and the medical specialist models, and in a few cases by the primary care model. HBOC and Lynch syndrome are typical examples of genetic disorders still managed principally by geneticists, although there is a progressive shift toward the involvement of other medical specialists. However, the clinical conditions mostly require the collaboration of several different specialists in a multidisciplinary team. Among other cost-effective approaches, FH cascade testing of relatives of index cases is delivered mainly through the medical specialist and the primary care models. This indicates that FH is mostly managed by primary care physicians, endocrinologists, or lipid specialists, and not necessarily by geneticists. The newborn screening panel, alongside BRCA1/2 screening among Ashkenazi Jews, is another cost-effective genetic testing service delivered via population-based screening programs.

Three critical findings stem from the review. First, some genetic programs, and the related delivery models that have been developed for the provision of the relevant genetic tests, lack sufficient evidence of clinical utility and validity and are currently not recommended for use in practice. The provision of these tests, classified as Tiers 2 and 3, could be related to faster genotyping technologies, the reduced cost of testing, commercial interests, and major public demand. It should be noted that these genetic programs comprise project proposals and demonstration projects (e.g., risk stratification models for genetic risk factors of common diseases) (Drury et al., 2007; Orlando et al., 2013), pilot studies (e.g., testing for various genetic conditions mainly for risk assessment purposes) (Moeschler et al., 2009; Bennett et al., 2010; Burton, 2012; Kirke et al., 2015), and integrated services (e.g., testing for surfactant dysfunction, skin cancer, or prostate cancer) (Henriksson et al., 2004; Epplein et al., 2005; Turcu et al., 2013). When considering proposals for full-scale projects, research ethics committees should approve only those studies on genetic tests with sufficient data on their validity and utility. On the other hand, pilot studies are undertaken to provide a preliminary assessment of benefit and to generate sufficient evidence to warrant a larger study. In this light, the results of pilot studies support the process of informed decision making and therefore could be justified for the assessment of genetic tests not yet included in Tier 1. Second, well-known medical journals and publishers have published the related studies on genetic tests with insufficient clinical data. Journals publishing medical genetics should consider adding the criteria that reported practices or interventions carried out in genetic services as full-scale projects should meet current evidence of efficacy and cost-effectiveness. Third, the percentage of studies reporting on informed consent prior to genetic testing was very low. The fact that consent forms were not reported in most studies may be ascribed to authors taking for granted the fact that informed consent is required prior to any medical intervention, since it is an important component of genetic counseling that assists patients in making informed decisions while prioritizing their healthcare needs, preferences, and personal, religious, and moral values. Thus, this finding does not necessarily indicate that informed consent is not routinely obtained in clinical practice, which would raise serious ethical and legal issues, but further research is warranted to clarify this issue.

### Limitations

The limits of the present study are related to restrictions in language and publication date, such that potentially relevant studies might have been excluded. However, most genetic tests were developed following the completion of the human genome sequencing in 2003 (Collins et al., 2003), justifying the choice of year 2000 as the lower date limit of the study. A critical point is the upper date limit (2015), which coincides with the first year of the PRECeDI project and which has not been updated. The present literature review is part of a multicenter European project that encompasses a systematic review of the literature in the first phase and a multicenter cross-sectional study in the second phase. The literature search was completed in 2015 and was followed by the development of online questionnaires for the multicenter cross-sectional study, which is currently ongoing and addresses European healthcare professionals with good knowledge of the provision of four selected genetic tests (BRCA1/2, Lynch syndrome, FH, inherited thrombophilia), of policies governing the provision of genetic testing and related services, and of the evaluation of genetic services (D'Andrea et al., 2016, 2018; Unim et al., 2016, 2017; Migliara et al., 2017; Rosso et al., 2017b; Tognetto et al., 2017; Di Marco et al., 2018; Pitini et al., 2018; Sénécal et al., 2018; Unim and Villari, 2018). The literature findings will be updated with the results of the multicenter cross-sectional study, when available, to incorporate new and relevant information.

Another limitation of the review concerns the adoption of the CDC evidence-based database of cost-effective genetic applications (Centers for Disease Control Prevention, 2018) for the classification of the genetic tests identified. The database does not comprise all possible genomic applications that could be classified using the level of evidence. However, it includes genetic tests identified in the present review and it is updated on a regular basis. Finally, due to the heterogeneity of the studies, a meta-analysis was not conducted; therefore, the results of the systematic review are presented as a narrative synthesis.

## Conclusion

In conclusion, the identification and evaluation of existing genetic service delivery models are important steps toward the enhancement and standardization of genetic service provision. Current delivery models, including the “classic” geneticists model, require the integration of genetics into all medical specialties, collaboration among different healthcare professionals, and the redistribution of professional roles. Prior to implementation in clinical and public health practice, genetic tests should be evaluated based on available efficacy and cost-effectiveness data and offered to citizens as a right to benefit from innovative healthcare. The proper implementation of genomics applications in mainstream medicine can be achieved through professional education, training, adequate funding, public policies, and public awareness of the field of genomic medicine.

It is advisable to evaluate the appropriate model for the provision of a genetic service with respect to the healthcare system and the genetic test provided within a specific genetic program, giving equal value to all elements in the program (i.e., genetic test, population target, clinical pathways, and overall organizational and economic aspects). To our knowledge this is the first study proposing a comprehensive classification of genetic service delivery models based on the role of healthcare professionals in service provision and patient care pathways. The present review may be useful in allowing clinical or public health practitioners and policy makers to identify current trends in the provision of personalized medicine.

## Author Contributions

BU and TL identified the studies through a literature search on online databases. BU, EP, TL, and GA performed the data extraction from the studies. PV, BU, CD, EP, and CM critically discussed and interpreted the results of the study. BU and PV wrote the manuscript with input from all authors. All authors read and approved the final manuscript. All authors contributed to the conception and design of the study.

### Conflict of Interest Statement

The authors declare that the research was conducted in the absence of any commercial or financial relationships that could be construed as a potential conflict of interest.
